# Layer-by-Layer Coatings of Collagen–Hyaluronic acid Loaded with an Antibacterial Manuka Honey Bioactive Compound to Fight Metallic Implant Infections

**DOI:** 10.1021/acsami.3c11910

**Published:** 2023-12-06

**Authors:** Anjaneyulu Udduttula, Nicholas Jakubovics, Imran Khan, Lucia Pontiroli, Kenneth S. Rankin, Piergiorgio Gentile, Ana M. Ferreira

**Affiliations:** † School of Engineering, Newcastle University, Newcastle Upon Tyne NE1 7RU, U.K.; ‡ School of Dental Sciences, Faculty of Medical Sciences, Newcastle University, Newcastle Upon Tyne NE1 7RU, U.K.; § Biomet UK Healthcare Ltd, Stella Building, Windmill Hill Business Park, Swindon SN5 6NX, U.K.; ∥ Translational and Clinical Research Institute, Faculty of Medical Sciences, Newcastle University, Newcastle upon Tyne NE2 4HH, U.K.; ⊥ Centre of Biomaterials, Cellular & Molecular Theranostics (CBCMT), Vellore Institute of Technology (VIT), Vellore, TN 632014, India

**Keywords:** jellyfish collagen, hyaluronic acid, methylglyoxal, layer-by-layer, antibacterial coating

## Abstract

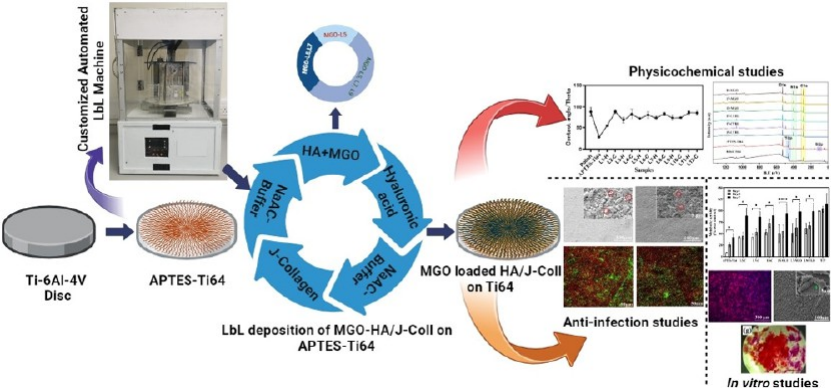

Implant-associated severe infections can result in catastrophic implant failures; thus, advanced antibacterial coatings are needed to combat infections. This study focuses on harnessing nature-inspired self-assembly of extracellular matrix (ECM)-like coatings on Ti alloy with a combination of jellyfish-derived collagen (J-COLL) and hyaluronic acid (HA) using our customized automated hybrid layer-by-layer apparatus. To improve the anti-infection efficacy of coatings, we have incorporated a natural antibacterial agent methylglyoxal (MGO, a Manuka honey compound) in optimized multilayer coatings. The obtainment of MGO-loaded multilayer coatings was successfully assessed by profilometry, contact angle, attenuated total reflectance (ATR)-Fourier transform infrared spectroscopy, scanning electron microscopy, and X-ray photoelectron spectroscopy. *In vitro* degradation confirmed the controlled release activity of MGO with a range of concentrations from 0.90 to 2.38 mM up to 21 days. A bacterial cell culture study using *Escherichia coli* (*E. coli*) and *Staphylococcus epidermidis* (*S. epidermidis*) confirmed that the MGO incorporated within layers 7 and 9 had a favorable effect on preventing bacterial growth and colonization on their surfaces. An *in vitro* cytocompatibility study confirmed that MGO agents included in the layers did not affect or reduce the cellular functionalities of L929 fibroblasts. In addition, MGO-loaded layers with Immortalized Mesenchymal Stem Cells (Y201 TERT-hMSCs) were found to favor the growth and differentiation of Y201 cells and promote calcium nodule formation. Overall, these surface coatings are promising candidates for delivering antimicrobial activity with bone-inducing functions for future bone tissue engineering applications.

## 1 Introduction

Implant-associated infections are a major concern in orthopedics. They can reduce implant function, lengthen the healing time, and cause implants to fail.^1^ These infections are often difficult to treat with antibiotics with potentially life-threatening consequences for patients and a severe economic burden.^2^ Extensive research has been directed toward improving the anti-infection ability of implants by different surface modification techniques including physical (etching, surface activation, ion implantation, etc.) and chemical (bioceramics, nanocomposites, polymeric coatings, etc.) approaches.^3,4^ In addition, implants have been coated with antibiotics^5^ such as vancomycin, penicillin, and gentamycin and/or inorganic antibacterial agents^6^ including silver, copper, gold, and zinc to reduce bacterial colonization and improve the performance of the implants. Furthermore, in recent years, a few studies have coated implants with antimicrobial peptides such as LL-37, HHC-36, GL13K, or bioabsorbable hydrogels to enhance the antibacterial activity of implant surfaces.^7^ However, some strategies for implant surfaces impregnated with antimicrobial agents involve complex manufacturing procedures and may be inefficient for combatting infections due to uncontrolled release, an effect on native tissue, and high costs to translate into clinical applications. Therefore, advanced strategies are needed for decreasing the incidence of infections and ensuring the long-term success of the implants. Our research groups have a strong background in the stimulation of bacterial resistance and osteogenic properties of implants by polyelectrolytic self-assembling coatings^8,9^ using our advanced hybrid layer-by-layer machine.

Titanium and its alloys (Ti-6Al-4V; Ti64) have been widely used in orthopedic and dental surgery due to their superior mechanical, corrosion-resistant, and biocompatible properties.^10^ For improving the implant properties by surface modification, the layer-by-layer (LbL) technique has gained popularity because it can mimic natural ECM microenvironments for the regeneration of tissue by enabling the self-assembly of oppositely charged polyelectrolytes with highly ordered three-dimensional (3D) complex structures *in vitro*. Also, matrices generated by LbL can incorporate small bioactive molecules such as peptides, growth factors, and antimicrobial agents into the polyelectrolytic multilayer (PEM) coatings.^11^ Previous works used the LbL technique to develop PEM coatings on Ti alloys using mammalian-derived collagen (COLL) and hyaluronic acid (HA) as cationic and anionic polyelectrolytes, respectively, to improve the biocompatibility and osteogenic induction abilities of implants. COLL is a naturally occurring protein in human connective tissues and plays a major role in the development of the ECM,^12^ whereas HA is a nonprotein glycosaminoglycan that occurs in the synovial fluid of cartilage.^13^ COLL and HA have been Food and Drug Administration (FDA) approved and widely used in different industrial applications including cosmetics and food.^14^ They can be easily adopted for clinical applications.

Some examples using dipping LbL include research work by Haiyong et al. that developed stable COLL/HA multilayer coatings on modified Ti alloy by a covalent immobilization-induced LbL technique and evaluated *in vivo* osseointegration performance for avoiding aseptic loosening of implants.^15^ Also, Qiaojie et al. studied the LbL stability of RGD and basic fibroblast growth factor (bFGF) biomolecules incorporated into a COLL/HA coating on Ti alloy and their effects on MC3T3-E1 cell growth and found early-stage healing ability *in vivo*.^16^ Chengzhong et al. fabricated COLL/HA multilayer coatings with grafting of BMP2/7 peptide molecules by the LbL process and compared its biological effects against BMP2, BMP7, and their mixture. They proved that PEM with BMP2/7 strongly promoted the differentiation of osteoblast cells.^17^ Ying et al. developed RGD-functionalized COLL/HA multilayer coatings on Ti by LbL and found these coatings rapidly influenced the attachment, proliferation, and differentiation of preosteoblast cells.^18^ Xiaojun et al. prepared LbL-developed COLL/HA PEM coatings on Ti and examined the effects on preosteoblast cells in terms of proliferation and differentiation.^19^ From the literature, it was found that the COLL/HA PEM has a tremendous response to bone regeneration and an easier fabrication process. All of these reports greatly suggest that COLL- and HA-based coatings on implants can induce better osteointegration function and improve the healing time of bone defects; however, these coatings do not possess any activity toward controlling infections. In addition, mammalian-derived COLL has drawbacks including immunogenicity, sustainability, and cost.^20^ Hence, this work explored the use of a more sustainable alternative material to mammalian COLL. As a novel approach, we used marine-derived jellyfish collagen (J-COLL) as a cationic PE, which is a bovine spongiform encephalopathy (BSE)-free and immunologically safer material. To the best of our literature survey, no reports exist on the use of J-COLL as PE for fabrication of multilayer coatings on Ti64 by the LbL process; hence, our work is intended to develop LbL coatings of J-COLL and HA on Ti64.

Moreover, the selection of an antimicrobial agent is a key factor in bone tissue engineering that should not compromise the osteogenic behavior of cells or effectiveness in the killing of drug-resistant bacteria. In this study, we explored the incorporation of methylglyoxal (MGO), which is a natural antimicrobial agent derived from Manuka honey. Also, this compound is a reactive dicarbonyl, produced during the metabolic fatty acid pathways and as a byproduct of glucose in the body.^21^ Through inhibition of mitochondrial respiration and glycolysis, MGO restricts bacterial growth and eradicates biofilm formation.^22^ Few reports exist on using MGO in combination with natural polymers for various applications such as combatting infections, wound dressings, fighting tumor cells, or regenerating connective tissues. Shaheer et al. fabricated MGO-conjugated chitosan nanoparticles and studied their effects on antimicrobial resistance in fungal infections.^23^ Adrita et al. used the Schiff base reaction to synthesize MGO-conjugated chitosan nanoparticles and found they acted as an immunostimulant, combatting the sarcoma-180 cells in tumors.^24^ Manni et al. developed bacterial cellulose and MGO nanocomposites and demonstrated antimicrobial activity against a broad spectrum of bacteria, suggesting that they could be useful for antimicrobial wound dressing materials.^25^ Amy et al. fabricated MGO-grafted COLL and studied effects on myofibroblast cells, which were found to undergo faster differentiation.^26^ However, there has been no research on MGO-integrated PEM coatings on implants by the LbL process and their anti-infection studies for bone regeneration application. Also, no reports have been delivered so far on the function of MGO in bone healing. Therefore, the novelty of our work is to develop MGO-loaded ECM-based J-COLL and HA molecules as layer-by-layer coatings on the Ti64 alloy using our customized automated hybrid LbL machine and study their anti-infection capabilities and bone-inducing activities.

In this study, multilayered coatings on Ti64 substrates based on J-COLL and HA polyelectrolytes with the incorporation of the MGO compound were manufactured by exploiting a patented hybrid LbL machine developed by Ferreira and Gentile at the Newcastle University, U.K. (US Patent 20220388024A1). The MGO-loaded HA/J-COLL-coated Ti64 were characterized by different analytical techniques to evaluate their physicochemical properties and examined for *in vitro* cytocompatibility using L929 cells and functionality via immortalized human mesenchymal stem cells (TERT-hMSCs, Y201). Furthermore, the antibacterial activity of MGO-loaded HA/J-COLL coatings against two key pathogens in implant-associated infections, *Escherichia coli* and *Staphylococcus epidermidis* was assessed by qualitative and quantitative techniques.

## 2 Materials and Methods

### 2.1 Materials

Ti-6Al-4V (Grade V; 20 mm × 2 mm) was purchased from Smith Metal Centre, Gateshead, U.K. 3-Aminopropyl triethoxysilane (APTES: 99%, Sigma-Aldrich, U.K.), methylglyoxal (MGO: 40% in H_2_O, Sigma-Aldrich, U.K.), sodium hyaluronate (HA: 750–1000 kDA, Contipro Inc. Czech Republic), and jellyfish collagen (J-COLL, 4 mg/mL concentration in 0.02 M acetic acid, Jellagen, U.K.) were used without further purification. All other commonly used chemical reagents, such as acids, bases, solvents, and buffer solutions, were purchased from Sigma-Aldrich (U.K.). Mouse-derived L929 fibroblast cells were purchased by Sigma-Aldrich (U.K.), while immortalized TERT-human mesenchymal stem cells (hMSCs) were developed at the University of York (Y201 line). The cell culture-related reagents, such as low glucose-Dulbecco’s modified eagle medium (LG-DMEM, phenol red free), fetal bovine serum (FBS), l-glutamine, and antibiotics, were purchased from Sigma-Aldrich (U.K.).

### 2.2 Surface Modification of Ti Alloys

#### 2.2.1 Acid–Alkali Treatment of Ti64 Substrates

Ti-6Al-4V discs (20 mm × 2 mm) underwent three polishing steps: silicon carbide papers (120–1200# grit), velvet cloth polishing (Struers LaboPol-5, Denmark), and finally distilled water (DD H_2_O) washing. The polished discs were subjected to ultrasonic cleaning with acetone for 15 min. To enhance the surface roughness and facilitate the formation of an oxide layer, the disc samples were immersed in a mixture of acids (H_2_SO_4_/HCl/H_2_O, 1:1:1 ratio) at 60 °C for 1 h. Next, the acid-treated samples were incubated in a 10 M NaOH solution at 60 °C for 24 h to promote hydrophilicity by the formation of Ti-OH layers. The discs were then washed with DD H_2_O and dried in an oven for 24 h at 45 °C.

#### 2.2.2 Silanization of Alkali-Ti64

To get the surface of the Ti64 samples to be activated with a positive charge, the alkali-Ti64 samples were treated with 10% APTES (pH 3) at 100 °C for 45 min and then dried with air in a fume hood for 24 h. The obtained APTES-Ti64 samples were further assessed for amine density using the acid orange II assay (Sigma-Aldrich, U.K.) by following the protocol of our previous work.^8^

### 2.3 Layer-by-Layer Self-Assembly of HA/J-Coll on APTES-Ti64

#### 2.3.1 Optimization of HA/J-Coll Layers

Based on our previous works on layer-by-layer coatings,^8,9,27^ we have determined the parameters, such as time and temperature, for our LbL deposition of HA/J-COLL on a Ti64 substrate. To obtain the LbL self-assembly of HA/J-COLL on APTES-Ti64, HA (1 mg/mL) and J-COLL (2 mg/mL) in a sodium acetate buffer (pH 5) solution were prepared by using an LbL automated machine. The positively charged APTES-Ti64 samples were first dipped into a negatively charged HA solution for 10 min, followed by a buffer wash for 5 min to develop the first layer. Subsequently, the second layer was deposited by being dipped into positively charged J-COLL solution for 10 min and then washed with the buffer for 5 min to remove the physically adsorbed molecules. Further, the abovementioned first and second layer deposition processes were repeated another five times to obtain the overall 12 layers. Finally, the fabricated HA and J-COLL self-assembled Ti64 were dried at room temperature and stored in a fridge to be used for further physicochemical characterization and biological studies.

#### 2.3.2 Incorporation of MGO into HA/J-Coll Layers

To incorporate the MGO antibacterial agent into layers, a mixture of solutions composed of negatively charged HA (1 mg/mL) and MGO (9 mM = 0.0625%) was prepared. Based on the optimization of HA and J-COLL layers on Ti64 samples, the fifth (L5), seventh (L7), and ninth (L9) layers were selected to incorporate the MGO agent. The loading of MGO increased with respect to these layers in terms of 1× (L5), 2× (L5, L7), and 3× (L5, L7, and L9) by following a similar protocol as described in Section 2.1, and controls were fabricated for the same number of layers without the addition of MGO.

### 2.4 Determination of MGO Quantification and Its Release Profile

Samples with 5, 7, and 9 layers forming the multilayered coating, with and without MGO, were put into a 12-well plate, and then 1 mL of DI H_2_O was added. The layers were solubilized in water by vigorously pipetting in and out several times, followed by incubation for 2 h under shaking conditions at room temperature. Then, a calorimetric-based MGO assay kit was used to determine the concentration of MGO in the coated samples. In this test, 20 μL of resulting solutions from L5 to L9 samples were transferred into a 96-well plate, and then 80 μL of MGO assay kit solution (Abcam, U.K.) was added and incubated for 2 h at room temperature. The MGO content in the layers was measured by using OD values at 450 nm using a microplate reader (BMG Labtech, FLUOstar Omega, Germany). Similarly, the MGO standard curve was built by preparing standard MGO solutions (0–0.5 mM) and mixing them with the MGO assay kit, followed by reading their absorbance values at 450 nm. The degradation of multilayers to MGO release from L5, L7, and L9 layers at different time points (1, 3, 7, 14, and 21 days) was carried out in phosphate-buffered saline (PBS) solution at 37 °C (Thermo Scientific, MIDI 40) and estimated using the abovementioned MGO quantification protocol.

### 2.5 Physicochemical Characterization

Optimized and MGO-incorporated HA/J-COLL-Ti64 were characterized by water contact angle, profilometry, attenuated total reflectance-Fourier transform infrared (ATR-FTIR) spectroscopy, scanning electron microscopy (SEM), and X-ray photoelectron spectroscopy (XPS) analyses. The ζ-potentials of HA, J-COLL, and MGO aqueous solutions used for coating were determined by injecting 800 μL of each into a folded capillary cell and placing it in a Zetasizer instrument (Malvern NANO ZS, U.K.). The functional groups of optimized and MGO-incorporated HA/J-COLL-Ti64 were determined by ATR-FTIR (Nicolet iN5, Thermo Fisher Scientific, U.K.) using 120 scans per min and a resolution of 2 cm^–1^ with a range of wavenumbers from 600 to 4000 cm^–1^. The surface morphology of optimized and MGO-incorporated HA/J-COLL-Ti64 was examined by SEM analysis (Jeol JSM-5600LV, U.K.) at 20 kV with support of gold sputtering of samples. The surface roughness was analyzed by a profilometer (Alicona SL-2014) with the measurements taken by a magnification of 5× lens, and the wetting behavior of optimized and MGO-incorporated substrates was observed by the liquid drop method (DD H_2_O) using a contact angle instrument (Rame-Hart instrument, Germany, 290-U1-170620) with a camera of German-MadeU4 Series. The surface composition of optimized and MGO-incorporated samples was determined by X-ray photoelectron spectroscopy (XPS; Theta Probe, Thermo Scientific, East Grinstead, U.K.). Survey spectra and high-resolution spectra were collected at a pass energy (200 and 40 eV), a step size (1 and 0.1 eV), and a dwell time (50 and 200 ms). The CasaXPS software was used to obtain the deconvolution contributions for high-resolution spectra.

### 2.6 Antibacterial Study of MGO-Loaded Samples

#### 2.6.1 Minimum Inhibitory Concentration (MIC) and Minimum Bactericidal Concentration (MBC)

*Escherichia coli* MG-3739 (*E. coli*) and *Staphylococcus epidermidis* NCTC-11047 (*S. epidermidis*) were used to study the antibacterial activity of MGO. *E. coli* was routinely cultured in Luria–Bertani broth (10 g/L, HIMEDIA, U.K.), while *S. epidermidis* was cultured in BHYE containing (per L, HIMEDIA, U.K.) 37 g of Brain Heart Infusion and 5 g of Yeast Extract. Different concentrations of MGO solutions (144 mM (1%), 72 mM (0.5%), 36 mM (0.25%), 18 mM (0.125%), 9 mM (0.0625%), 4.5 mM (0.0312%), 2.25 mM (0.0156%), and 1.125 mM (0.0075%)) were prepared by dilution with respective pathogen growth media (LB-*E. coli* and BHYE-*S. epidermidis*). The two separate sets of growth medium (4 mL) containing falcon tubes with different concentrations of MGO were inoculated with 2.5 μL of *S. epidermidis* and *E. coli* stock pathogen solutions. The *E. coli* inoculated samples were placed in a shaking incubator (180 RPM) at 37 °C, whereas *S. epidermidis* samples were kept at 37 °C in an incubator without shaking for 24 h. After the incubation period, both pathogen-treated samples were visually checked for the growth of bacteria, and their O.D. values were collected. To determine the MBC of MGO, aliquots of 150 μL of MIC-detected samples with no visible bacterial growth of *E. coli* and *S. epidermidis* were subcultured onto LB and BHYE plates, respectively, and incubated overnight at 37 °C.

#### 2.6.2 Planktonic and Biofilm Quantification by the Spread Plate Method and Colony Forming Unit (CFU) Analysis

The APTES-Ti64, control, and MGO-loaded samples with L5, L7, and L9 layers were sterilized by placing them under a UV lamp at a wavelength of 240 nm for 30 min on each side. Then, samples were placed in a 12-well plate with the coating area exposed to the outside. To the samples, 1 mL of *E. coli* and *S. epidermidis* bacterial suspension containing 1 × 10^6^ CFU (0.1 OD) was added and incubated for 24 h at 37 °C. Samples were then diluted from 10^–1^ to 10^–8^, and triplicate 20 μL drops were placed on an agar plate and allowed to dry. After incubation at 37 °C overnight, dilutions that produced between 10 and 100 colonies were selected, and colonies were counted. A similar method was performed to enumerate viable bacteria in biofilms, except that an extra step was included to detach bacteria from the surface of samples using cell scrapers.

#### 2.6.3 Bacterial Growth Assessment by Live/Dead Staining

The APTES-Ti64, control, and MGO-loaded samples were treated with 1 mL of growth medium containing *E. coli* and *S. epidermidis* and were incubated for 24 h at 37 °C, followed by washing three times with PBS. Dyes were prepared by adding Syto-9 (1.5 μL) and propidium iodide (PI, 1.5 μL) reagents (LIVE/DEAD BacLightTM Bacterial Viability Kit, Thermo Fisher, U.K.) to 1 mL of PBS. One mL of Syto-9 and PI mixture was added to the samples and stored for 15 min in the dark, followed by rinsing twice with PBS. Finally, the samples were inverted in wells with PBS to avoid drying and observed under a confocal laser scanning microscope (CLSM, Zeiss LSM800, U.K.).

#### 2.6.4 Bacterial Adhesion by SEM Analysis

The APTES-Ti64, control, and MGO-loaded L5, L7, and L9 layers were cultured with *E. coli* and *S. epidermidis* microbes for 24 h of incubation as described above. Samples were fixed by incubation with 2.5% glutaraldehyde overnight at 4 °C. Samples were dehydrated by washing with a gradient series of ethanol solutions up to 100% and dried at the critical point of CO_2_. Samples were sputter-coated with gold and observed under SEM (Tescan Vega 3LMU, U.K.).

### 2.7 *In Vitro* Cell Culture Study Using L929 Fibroblast Cells

Mouse-derived L929 fibroblast cells were used to assess the cytocompatibility of APTES-Ti64, control, and MGO-loaded L5, L7, and L9 samples via metabolic activity assays (PrestoBlue Cell viability Reagent, Invitrogen, Thermo Fisher, U.K.), live/dead assay (ReadyProbes Cell viability imaging kit, Blue/Green, Invitrogen, Thermo Fisher, U.K.), and cytoskeleton staining (ActinRed 555 ReadyProbes reagent, Invitrogen, Thermo Fisher, U.K. and Fluoroshield with DAPI, Sigma-Aldrich, U.K.). All sterilized samples were placed in a 12-well plate, followed by the addition of 200 μL of DMEM medium with 30,000 cells and gently spread on the surface of the samples. To the cells with samples, we added 1 mL of DMEM medium (10% FBS, 1% penicillin/streptomycin) as a growth medium and incubated at 37 °C in 5% CO_2_ for 1, 3, and 7 days.

#### 2.7.1 PrestoBlue Assay

The medium was removed from the samples at the end of each incubation period and washed twice with PBS. Then, 1 mL of 10% PrestoBlue solution (1 mL of PrestoBlue in 10 mL of PBS) was added to each sample and incubated for 2 h and protected from light. Then, 100 μL of metabolized solution was transferred from each well to a 96-well plate, and the fluorescence was measured at 480/590 nm by fluorescence spectroscopy (BMG Labtech, FLUOstar Omega, Germany). All of the experiments were performed independently three times, and the obtained values were normalized using a tissue culture plate (TCP) and control values.

#### 2.7.2 Live/Dead Assay

The live/dead assay was employed to study the cell viability of L929 cells at 1 and 7 days under different sample conditions. The samples were washed with PBS solution, followed by the addition of a mixture of NucBlue (live cell staining) and NucGreen (dead cell staining) reagents (2 drops of each reagent per 1 mL of PBS as per the protocol of Invitrogen, Thermos Fisher, U.K.). Then, the samples were incubated for 15 min at 37 °C in 5% CO_2_ and observed under fluorescence microscopy (Invitrogen EVOS-M5000, U.K.).

#### 2.7.3 Cytoskeleton Staining

On day 7, the medium was removed from the different samples and washed with PBS, followed by fixing the cells by adding 4% paraformaldehyde and placing them at 4 °C overnight. Then, the samples were washed with PBS twice, and ActinRed solution was added (2 drops in 1 mL of PBS + 0.1% Tween) and incubated at room temperature for 30 min to stain the cellular cytoskeleton. The samples were washed in PBS solution, followed by the addition of one drop of DAPI solution directly on the surface of the samples and stored for 10 min at room temperature. Finally, the samples were imaged by fluorescence microscopy (Invitrogen EVOS-M5000, U.K.).

#### 2.7.4 SEM Analysis

At day 7, the cell medium was removed from the different samples and washed with PBS, followed by fixing the cells by adding 2.5% glutaraldehyde and storing them overnight at 4 °C. Then, the samples were dehydrated by a series of ethanol solutions (70, 80, 90, and 100%), followed by critical point drying and gold sputtering for SEM analysis (Tescan Vega 3LMU, U.K.).

### 2.8 *In Vitro* Cell Culture Study Using Y201 MSCs

#### 2.8.1 Cytoskeleton Staining and SEM Analysis

The UV-sterilized APTES-Ti64, control, and MGO-loaded samples were placed in a 12-well plate, and their surfaces were seeded by Y201 TERT-hMSCs with a density of 2 × 10^5^ cells per substrate. Further, the samples were incubated for 7 days at 37 °C with interval refreshment of DMEM growth medium. At day 7, the samples were washed twice with PBS solution, and 4% paraformaldehyde was added to fix the cells, followed by storage at 4 °C. The cytoskeleton staining and SEM analysis were performed by following the protocol described previously.

#### 2.8.2 Alizarin Red Staining Assay

To assess the calcium deposition by Y201 TERT-hMSC cells after 7 days of incubation, the samples were treated with an Alizarin Red staining solution. For this, samples were washed twice with DD H_2_O, followed by the addition of 1 mL of Alizarin Red staining solution and stored at room temperature for 30 min. After staining, the samples were washed with DD H_2_O several times until the water was clear and then imaged in a Brightfield (Leica LED5000 SLI microscope, U.K.).

### 2.9 Statistical Analysis

For every sample, tests were run at least three times independently. The mean and standard deviation were used to represent the results. The statistical analysis, which considered Parametric One-Way Analysis of Variance on Ranks (ANOVA) with Turkey and Dunnett posthoc test for the statistical differences between the groups and compared with control, was conducted using GraphPad Prism 9. Differences were considered statistically significant (*) at *p* < 0.05. Significance levels (*p* < 0.001) were indicated where appropriate (**).

## 3 Results

### 3.1 Assessment of J-Collagen, Hyaluronic Acid, and MGO Charge via ζ-Potential

**Figure 1:**
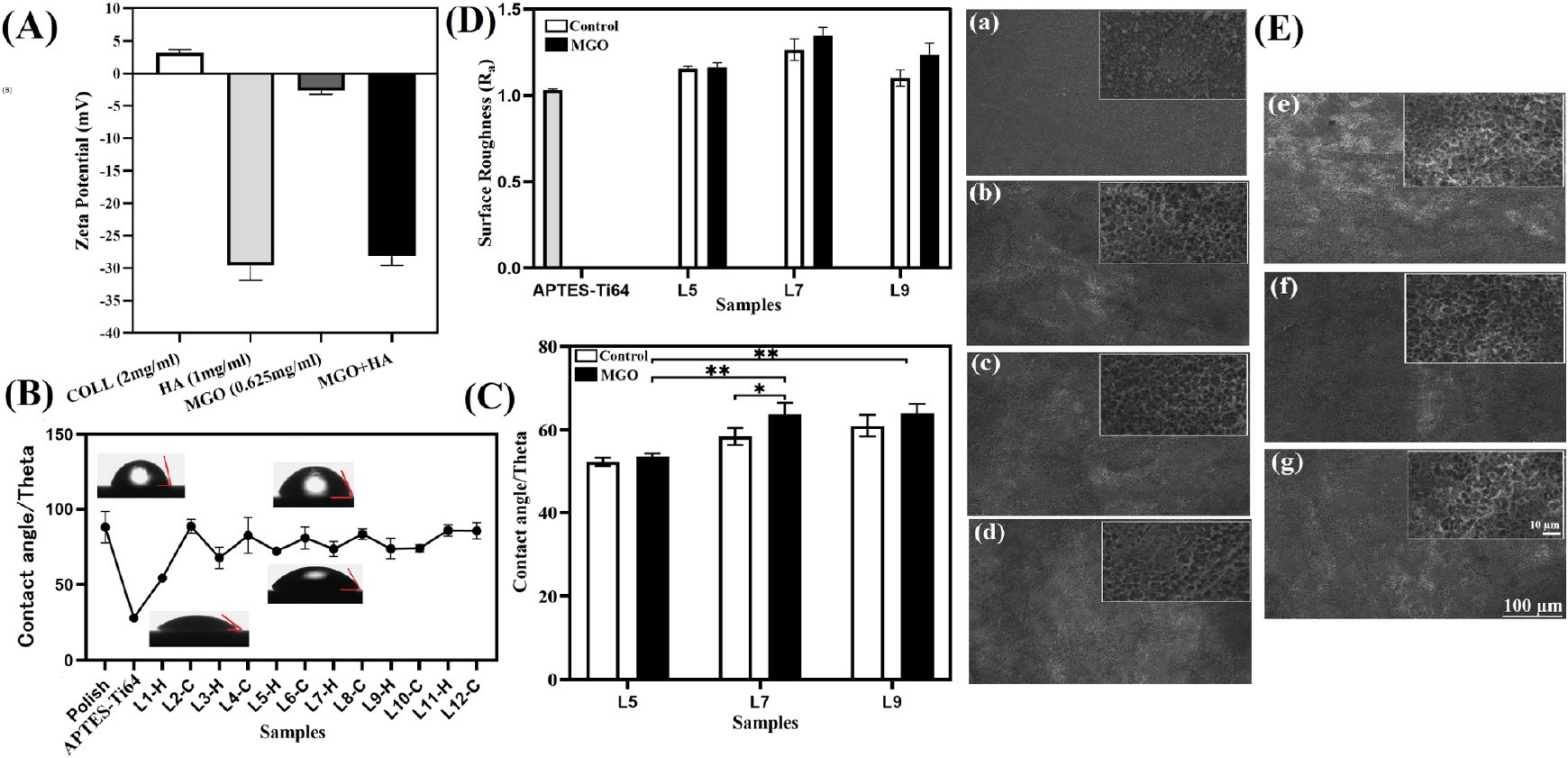
(A) ζ-Potential results of collagen, hyaluronic acid, MGO, and HA–MGO mixture; contact angle analysis of (B) optimized HA/COLL layers, (C) MGO-incorporated L5, L7, and L9 layers; (D) profilometry analysis of APTES-Ti64, control, and MGO-incorporated L5, L7, and L9; (E) SEM images of (a) APTES-Ti64, control, and MGO-incorporated L5 (b, e), L7 (c, f), and L9 (d, g); scale bars correspond to 100 μm and inset image’s scale bar corresponds to 10 μm.

The surface charges of J-COLL, HA, MGO, and HA–MGO were measured by a ζ-potential instrument using a sodium acetate buffer (pH 5), and the data are shown in Figure 1A. The J-COLL (2 mg/mL) had a weak positive charge (ζ = +3.11 mV), while HA (1 mg/mL) exhibited a strong negative charge (ζ = −29.36 mV) on its surface. MGO (0.625 mg/mL) was found to be a negatively charged compound (ζ = −3.08 mV); hence, we prepared a simple mixture of solution by addition of HA since it is negatively charged for further incorporation of MGO into a multilayer system of HA/J-COLL on Ti64. The surface of Ti64 substrates was activated with positively charged ammonium ions by silanization to allow the layer-by-layer self-assembly of oppositely charged polyelectrolytes, and the amount of amine group deposition was assessed by the Acid Orange test, as shown in Figure S1A,B. The amine density (109 μM/cm^2^) was found to be significantly higher for APTES-Ti64 than for polished and alkali-treated Ti64.

### 3.2 Assessment of Coated Ti64 Substrates Wettability via Contact Angle Analysis

The contact angle data in Figure 1B represent the layer-by-layer deposition of HA and J-COLL on Ti64. From these data, it was observed that the polished Ti64 exhibited hydrophobic behavior with a higher water contact angle (WCA) of 88.2°. The APTES-treated Ti64 showed a wettable surface with WCA 28.1° due to surface attachment and condensation of positively charged ammonium ions (NH_4_^+^) with the silane network.^28,29^ Next, the first layer deposition displayed a slightly higher WCA value of 54.5° than that of APTES-Ti64, which indicates the presence of an HA layer with good hydrophilicity, while the second layer has a hydrophobic nature with 88.7°, which denotes deposition of a J-COLL layer. According to the literature, COLL type I is hydrophobic with WCA values of 110°, while HA has a hydrophilic nature with associated values around 30°.^30^ Subsequent deposition of the third layer showed a lower WCA value of 67.8°, which indicates the initiation of the self-assembly of HA and J-COLL via electrostatic interactions. From the fourth to the ninth layer, WCA values present a typical LbL “zigzag” trend as per the oppositely charged HA and J-COLL PEs, suggesting a successful buildup of multilayers. Finally, the layers from 9 to 10th and 11 to 12th have a similar trend of WCA behavior due to the physical adsorption of HA and J-COLL on each other. Therefore, from these WCA results, we confirmed that, up to the ninth layer, effective self-assembly occurred between HA and J-COLL, and the coatings were stable with a good hydrophilic nature.

#### 3.2.1 Incorporation of MGO into HA/COLL-Ti64

HA and J-COLL self-assemblies and their stable multilayer deposition were optimized up to the ninth layer. Further, based on the effective built-up layers, we have selected the fifth, seventh, and ninth layers to incorporate the Manuka honey-derived antibacterial MGO compound. Figure 1C displays the WCA values of control and MGO-incorporated samples; from these data, it can be observed that MGO-added L5, L7, and L9 have a slight increase in WCA values when compared to control samples due to the incorporation of the MGO compound within the HA layer and the change in the surface topography. Also, significant differences in WCA values were observed from L5 to L9 with the inclusion of MGO. This WCA result confirmed that MGO does not have an adverse influence on the hydrophilic nature of HA/J-COLL multilayer coatings on Ti64.

### 3.3 Morphological Analysis of Coated Ti64 Substrates via Profilometry Analysis

The surface roughness of control and MGO-incorporated L5, L7, and L9 samples was further assessed by surface topography profilometry (Figure 1D). The APTES-Ti64 exhibited roughness values (*R*_a_) of 1.037 μm, which from similar works^31^ can be attributed to the formation of a silanization network. The control samples such as L5, L7, and L9 had no statistically significant differences among their surface roughness values (*R*_a_ = 1.1032–1.2658 μm) due to uniform surface deposition of layers and porous structures. The MGO incorporating L5, L7, and L9 displayed nonstatistically significant differences (*R*_a_ = 1.1636–1.3466 μm) when compared to controls. This behavior denotes the well-ordered accumulation of MGO into L5, L7, and L9 layers with no influence on the surface roughness. The change in surface roughness from APTES to control and MGO-included samples represents deposition of multilayers on Ti64. The profilometry two-dimensional (2D) and 3D images (Figure S2A,B) of control and MGO-loaded multilayers were found to be substantiated by the statistical results of surface roughness.

### 3.4 Morphological Analysis of Coated Ti64 Substrates via SEM Analysis

Figure 1E shows SEM images of APTES-Ti64, control, and MGO-included samples. From SEM micrographs, it was observed that the APTES-Ti64 disc surface exhibits a porous nature with minimal cracks. The control and MGO-incorporated samples displayed highly porous structures, and the pores were well-interconnected with the accumulation of polyelectrolyte molecules. However, with the increased number of layers from L5 to L9 for control and MGO samples, the dense nature of coatings was found to increase when compared with APTES-Ti64.

### 3.5 Chemical Analysis of Assembled HA/J-COLL Coating onto Ti64 via XPS Analysis

**Figure 2:**
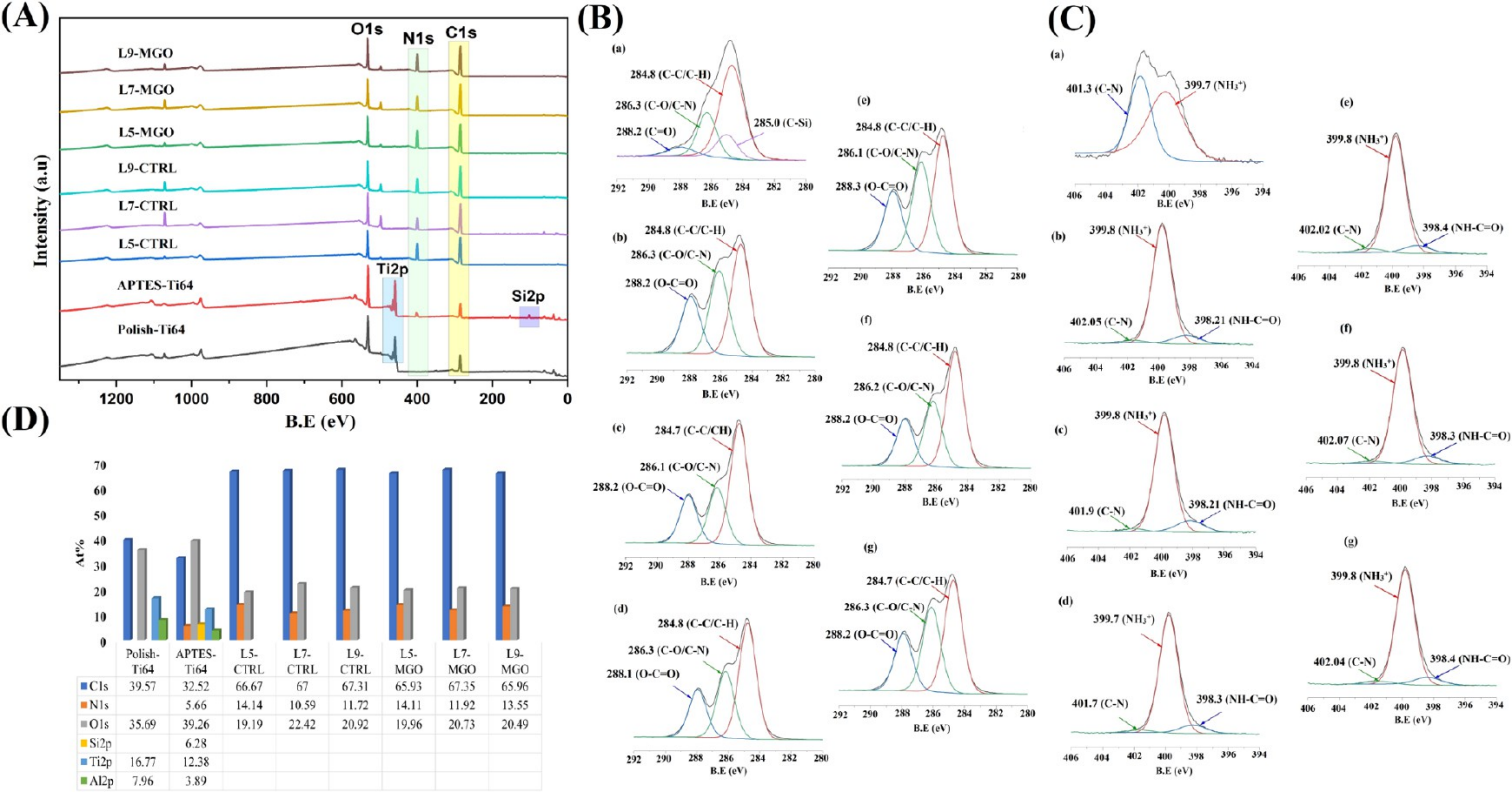
XPS analysis of APTES-Ti64, control, and MGO-incorporated L5, L7, and L9. (A) Survey spectra, (B, C) deconvolution spectra for C 1s and N 1s, and (D) elemental composition.

Polished, APTES, control, and MGO-incorporated L5, L7, and L9 samples were analyzed for their incidence of surface chemical states of elements and their concentrations by XPS analysis, which is a surface-sensitive quantitative method. From XPS results (Figure 2A–C), the polished Ti64 showed peaks at 530.6, 458.5, 284.8, and 74.9 eV, which correspond to O 1s, Ti 2p, C 1s, and Al 2p elements, whereas APTES-Ti64 had characteristic peaks for N 1s and Si 2p at 401.2 and 101.9 eV, respectively. These peak positions confirmed the surface activation of Ti64 with APTES treatment by the inclusion of ammonium ions (C–NH_4_^+^) and a silica network (C–Si–O). The control samples such as L5, L7, and L9 exhibited distinctive peaks at 400.0 and 285.1 eV, which were assigned to N 1s and C 1s, representing HA and J-COLL because both polyelectrolytes have major content of N and C elements.^8^ However, we found that the relative peak intensities of N 1s and C 1s were significantly increased compared with APTES and Polish-Ti64 samples, implying deposition of HA/J-COLL multilayers on Ti64 samples. Also, the peaks for Ti 2p, Al 2p, and Si 2p disappeared in control samples due to the buildup of HA/J-COLL coatings with thickness. The XPS spectra for MGO including L5, L7, and L9 samples showed similar characteristic peaks as control samples, with no significant evidence observed for MGO due to its dicarbonyl compound nature and lower-concentration incorporation into multilayer coatings. The atomic concentrations of polish, APTES, control, and MGO-included samples were reported in Figure 2D. The polish-Ti64 has Ti 2p (16.8%), Al 2p (7.9%), C 1s (39.67%), and O 1s (35.7%), whereas APTES-Ti64 has additional elemental concentrations such as N 1s (5.7%) and Si 2p (6.3%). The control and MGO-included samples showed comparable C 1s and N 1s concentrations from L5 to L9 in the ranges of 65.9–67.3 and 10.6–14.1%.

### 3.6 Chemical Analysis of the Assembled HA/J-COLL Coating onto Ti64 via ATR-FTIR Spectroscopy

**Figure 3:**
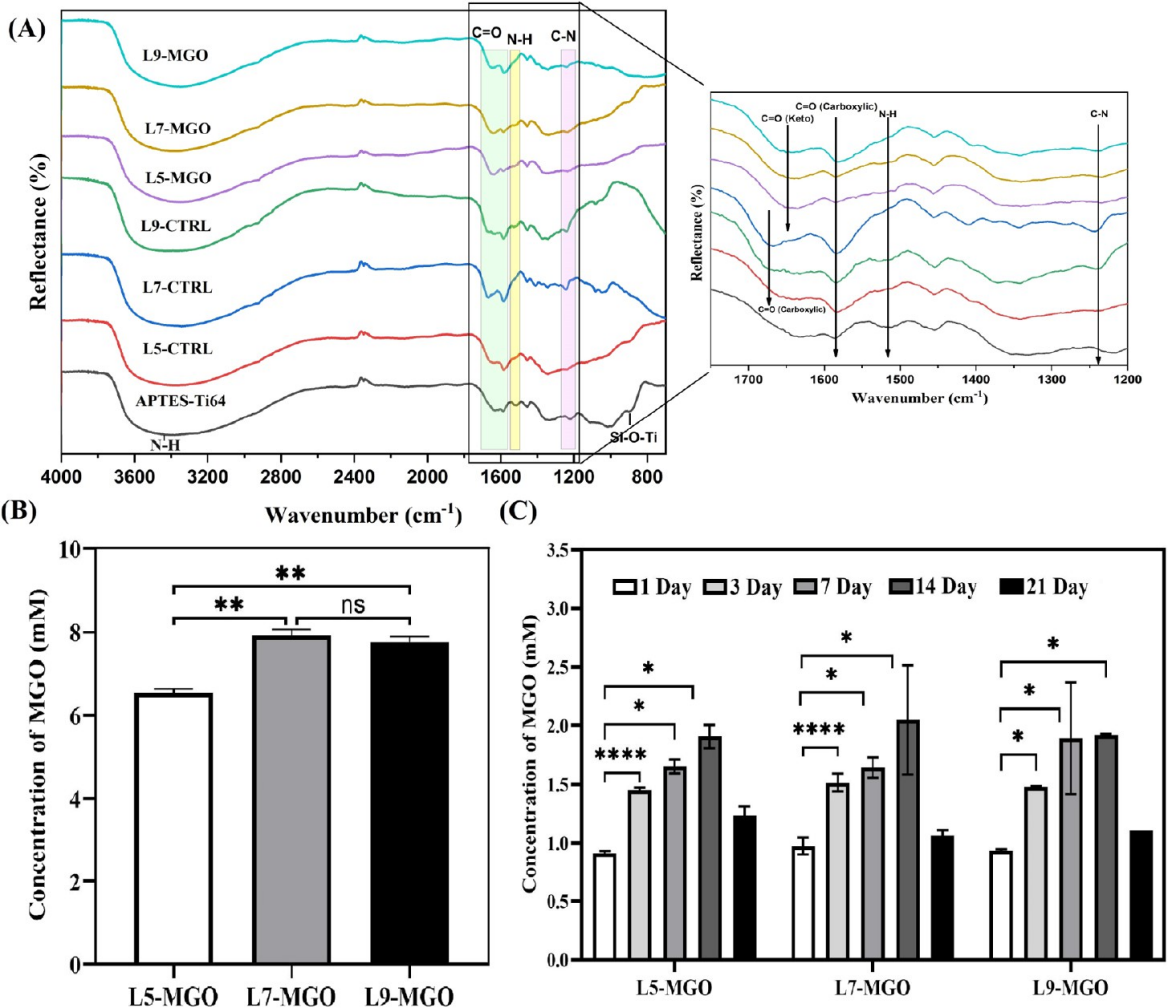
(A) ATR-FTIR analysis of APTES-Ti64, control, and MGO-incorporated L5, L7, and L9; (B) Estimation of MGO loading efficacy into L5, L7, and L9 layers and (C) MGO release (in mM) from coatings at different time points.

The chemical composition and functional groups of APTES, control, and MGO-incorporated samples were assessed by ATR-FTIR spectroscopy, as shown in Figure 3A. From ATR-FTIR spectra, the APTES-Ti64 exhibited characteristic absorption bands at 893, 1346, 1588, 1634, and 3432 cm^–1^, which indicate the presence of stretching modes of Si–O–Ti, C–N, bending vibration of N–H, bending mode of O–H (OH^–^), and stretching of N–H bonds (NH_3_^+^), respectively.^32^ It is apparent that HA and J-COLL polyelectrolytes combined provide a distinctive FTIR spectrum that has some notable peaks in a similar band range for each of the individual components. The typical characteristic bands at 1648, 1525, and 1232 cm^–1^ are attributed to C=O (amide-I), N–H (amide-II), and C–N (amide-II) groups in J-Coll. However, HA’s existence is confirmed by the peak position at 1584 cm^–1^ (C=O) of the carboxylic acid group. It was noticed that all control samples (L5, L7, and L9) displayed an intensified peak at 1584 cm^–1^ (C=O) due to the presence of the outermost layer of HA, and with an increase in J-COLL layer deposition, the peak position was slightly shifted to 1674 cm^–1^ (C=O, amide-I), which indicates the electrostatic deposition of oppositely charged J-Coll on HA.^8,33,34^ The vibrational band at 1241 cm^–1^ corresponds to the stretching mode of C–N groups in the amides of HA/J-COLL multilayers; the intensity increased with an increase in the layers from L5 to L9. The MGO-included samples exhibited similar ATR-FTIR spectra as control samples; however, it was obviously noted that a strong distinctive band occurred at 1644 cm^–1^ where it was not identified in the control samples. Also, this vibrational peak position was found to be slightly shifted toward a lower wavenumber due to the existence of the carbonyl group (C=O) in the MGO compound.^35^ In contrast to control samples, the peak intensity at 1584 cm^–1^ was slightly minimized due to the mixture of MGO and HA. These ATR-FTIR spectra confirmed the presence of the MGO compound in the multilayers of HA/J-COLL-Ti64.

### 3.7 Assessment of MGO Compound and Its Release from HA/J-COLL-Ti64

#### 3.7.1 Quantification of MGO Concentration

The colorimetric-based MGO assay kit was used to estimate the MGO concentration in MGO-incorporated L5, L7, and L9 samples, with results presented in Figure 3B. From an MGO standard curve, we determined the unknown concentration of MGO incorporated in each multilayered coating composed of 5 (L5), 7 (L7), and 9 (L9) layers, which were in the range of concentrations from 6.60 to 7.82 mM. These concentrations of MGO from multilayers were in good correlation with the theoretical MGO concentration added into multilayer coatings (9 mM) during the fabrication of HA/COLL LbL coatings on Ti64, with L5 showing a significantly lower amount of MGO compared to L7 and L9.

#### 3.7.2 Releasing Profile of MGO from Assembled HA/J-COLL Coating onto Ti64

The releasing behavior of MGO content from L5, L7, and L9 layers was quantitatively determined by an MGO assay kit at 1, 3, 7, 14, and 21 days, as shown in Figure 3C. From the release profile, it was noticed that MGO-loaded L5, L7, and L9 samples exhibited a gradual release of MGO content (0.90–2.38 mM) from their layers with an increase in the incubation period from 1 to 14 days, while at day 21, all samples demonstrated a lower concentration of released MGO (1.30 mM). The MGO-loaded layers exhibited a significant difference in releasing activity among their incubation points, an evidence that MGO is released from layers in a controlled manner throughout the incubation period.

### 3.8 *In Vitro* Assessment of MGO-Loaded HA/J-COLL Coating Impact on Bacterial Activity

#### 3.8.1 Assessment of Antibacterial Activity of the MGO Compound at Different Concentrations

The conventional microbiological tests, MIC, and MBC assays were used to assess the antimicrobial activity of the MGO compound against *E. coli* and *S. epidermidis* bacteria. From the MIC assay (Figure S3B), it was found that *E. coli* (Figure S3A-i) and *S. epidermidis* (Figure S3A-ii) bacteria were completely inhibited at MGO concentrations of 2.25 mM (0.0156%) and 9 mM (0.0625%), respectively. Also, similar concentrations showed killing efficacy in MBC assays (Figure S3Ci-ii), as observed in similar works.^36^

#### 3.8.2 Planktonic and Biofilm Quantification

**Figure 4:**
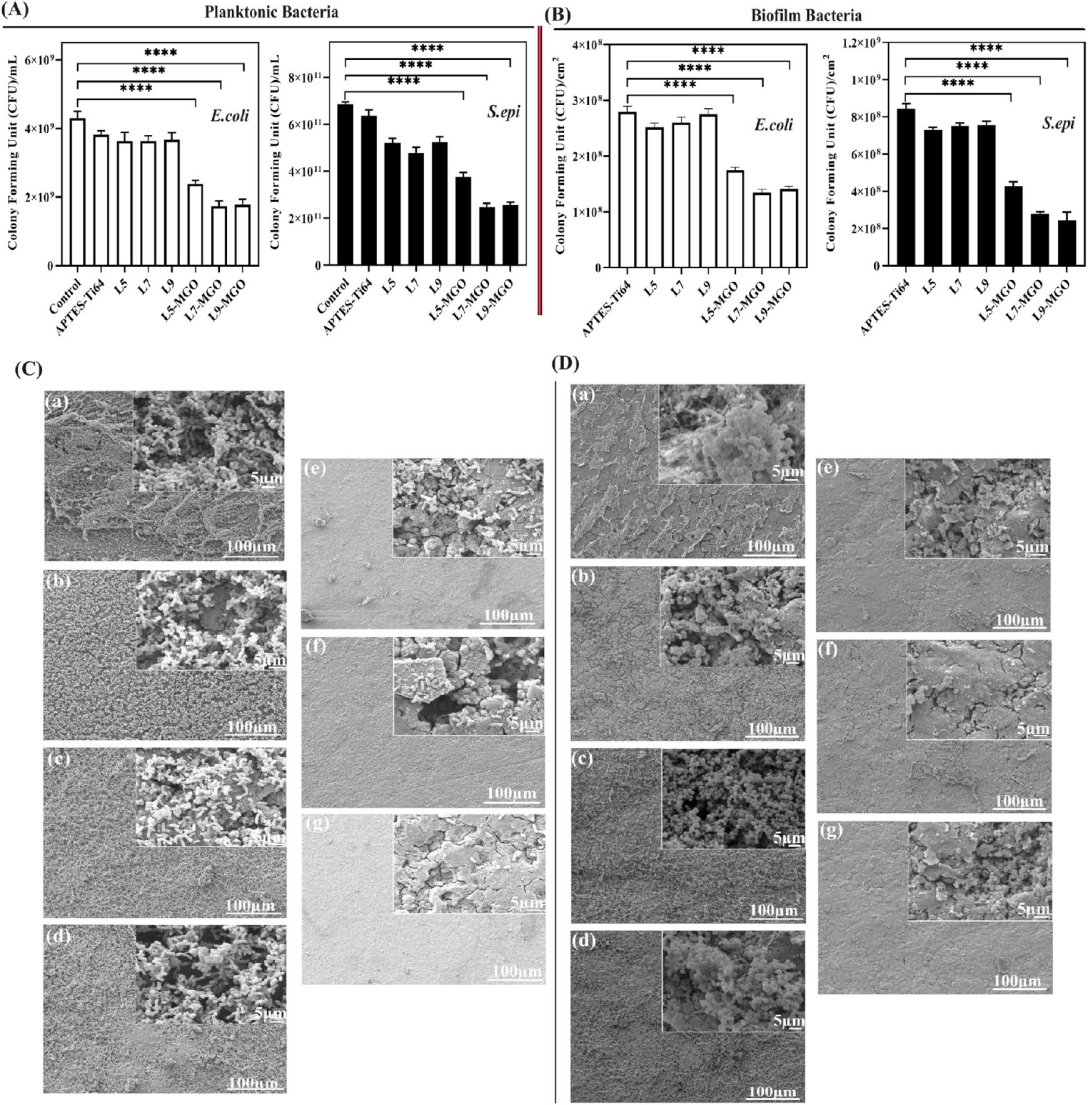
(A) Planktonic bacteria and (B) biofilm bacteria quantification of control and MGO-incorporated HA/COLL LbL-coated Ti64 using *E. coli* and *S. epidermidis* for 24 h by the CFU method; SEM images of (C) *E. coli* and (D) *S. epidermidis* bacteria on the surface of control and MGO-incorporated layers: (a) APTES-Ti64, (b) L5, (c) L7, (d) L9, (e) L5-MGO, (f) L7-MGO, and (g) L9-MGO. (*p < 0.05, **p < 0.01, ***p < 0.001, ****p < 0.0001). Scale bars correspond to 100 μm and inset image’s scale bar is 5 μm.

*In vitro* evaluation of the bactericidal property of MGO-incorporated L5, L7, and L9 samples was assessed by qualitative (SEM and CLSM) and quantitative (CFU) methods by inhibiting planktonic colonization and biofilm formation of bacteria. APTES-Ti64, control, and MGO-layered samples treated with both bacteria for 24 h were serially diluted, and colonies were quantified. Dilutions of 10^–6^ and 10^–8^ were selected for counting *E. coli* and *S. epidermidis*, respectively, as shown in Figure S4a,b. The planktonic viability of *E. coli* and *S. epidermidis* was determined for APTES-T64, LbL control, and MGO-loaded samples, and the results are shown in Figure 4A. From planktonic quantification results, it was observed that MGO-loaded layers of L5, L7, and L9 showed significantly less viability of both bacteria than the control layered samples. Among the MGO-layered samples, the L7 and L9 layers with MGO exhibited better efficacy at preventing bacterial colonization than the MGO-loaded L5 layer. However, both MGO-loaded L7 and L9 showed similar bactericidal activity with no significant difference between them. This inhibition of planktonic *E. coli* and *S. epidermidis* confirms that the MGO could be released from the layers and exhibit the consequent killing behavior on bacteria. Also, the biofilm bacterial growth on the surface of APTES-Ti64, control, and MGO-loaded layers was investigated (Figure 4B). Dilutions in the range of 10^–5^ for both bacterial samples were selected for counting (Figure S5a,b). From Figure 4B, it was found that the colonies of *E. coli* and *S. epidermidis* biofilm bacteria showed a trend analogous to that of planktonic bacteria. The viability of biofilm bacteria was found to be significantly reduced for MGO-L7 and L9 layers and higher for APTES-Ti64 and control samples. From planktonic and biofilm quantification results, the MGO content at an increased concentration in layers 7 and 9 has a favorable effect on inhibiting bacterial growth and colonization on their surfaces.

#### 3.8.3 Morphological Analysis of *E. coli* and *S. epidermidis* Bacterial Adhesion via SEM Analysis

*E. coli* and *S. epidermidis* bacterial adhesion and their morphological features on the surface of APTES-Ti64, control, and MGO-L5, L7, and L9 samples were examined by SEM analysis and micrographs illustrated in Figure 4C,D. *E. coli* (Figure 4C) and *S. epidermidis* (Figure 4D) treated samples exhibited rod- and spherical-shaped cell deposition, respectively. Extensive biofilms were formed on the surfaces of APTES-Ti64 and control samples without any obvious damage or disruption of bacterial cell integrity. Although MGO-incorporated layered surfaces displayed reduced bacterial growth of *E. coli* and *S. epidermidis* with impairment of the cell structure, it was observed that no significant difference among MGO-loaded samples was observed for both bacterial cells. These images confirmed that HA/J-COLL layers with the integration of MGO could strongly inhibit bacterial colonization and biofilm formation.

#### 3.8.4 Assessment of MGO-Loaded HA/J-COLL Coating Impact on Bacterial Viability

**Figure 5:**
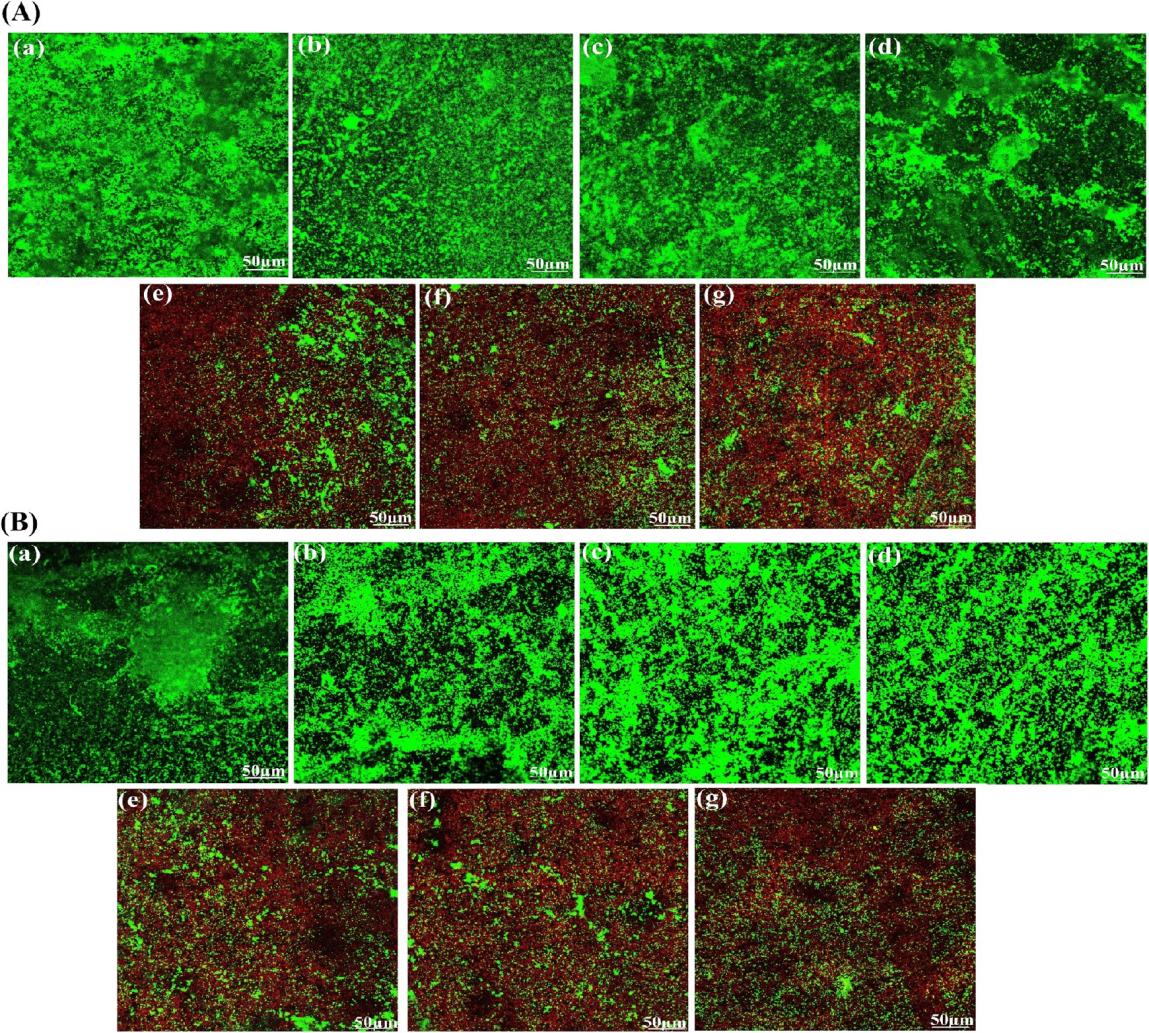
Live/dead staining of (A) *E. coli* and (B) *S. epidermidis* bacteria on the surface of control and MGO-incorporated layers for 24 h: (a) APTES-Ti64, (b) L5, (c) L7, (d) L9, (e) L5-MGO, (f) L7-MGO ,and (g) L9-MGO (green―live and red―dead bacteria). Scale bar: 50 μm.

The live and dead bacterial staining was used to examine *E. coli* and *S. epidermidis* colonization on the surfaces of APTES-Ti64, control, and MGO-loaded samples by CLSM analysis. The confocal laser scanning microscopy (CLSM) results are represented in Figure 5A,B. From CLSM images, it was observed that *E. coli* (Figure 5A) and *S. epidermidis* (Figure 5B) bacterial cells were well-colonized and developed as biofilms on the surface of the APTES-Ti64 and control samples. The surfaces of all APTES-64 and control samples were completely occupied with green color due to the existence of live bacterial cells. In contrast, the surfaces of the MGO-loaded L5, L7, and L9 samples had intense red staining, reflecting high levels of cell damage. The red color intensity increased for MGO-loaded L7 and L9 samples compared with L5, likely due to an increase of the MGO content in the additional layers, resulting in higher death of both strains (quantification shown in Figure S6). From CLSM results, it appears that MGO-incorporated sample surfaces could minimize the growth of cells and were favorable for contact-killing of both bacterial strains.

### 3.9 *In Vitro* Assessment of MGO-Loaded HA/J-COLL Coating on Cellular Behaviors

**Figure 6:**
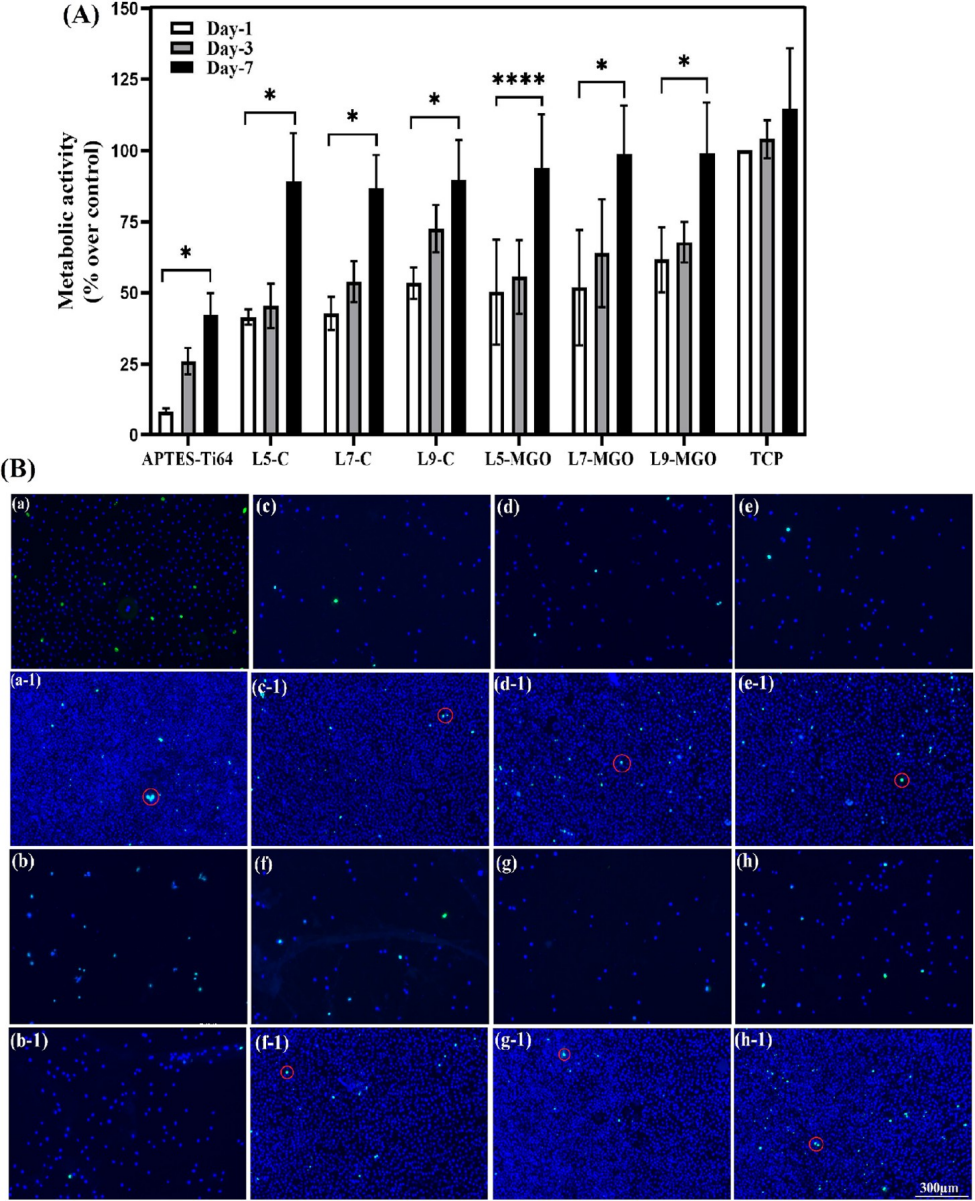
(A) PrestoBlue assay analysis of control and MGO-incorporated L5, L7, and L9 samples for 1, 3, and 7 days using L929 fibroblast cells; (B) Live/dead staining of (a) blank, (b) APTES-Ti64, (c–e) control, and (f–h) MGO-incorporated L5, L7, and L9 at 1 (a–h) and 7 (a-1 to h-1) days; scale bar: 300 μm (live cells = blue, dead cells= green/highlighted in red circles).

**Figure 7:**
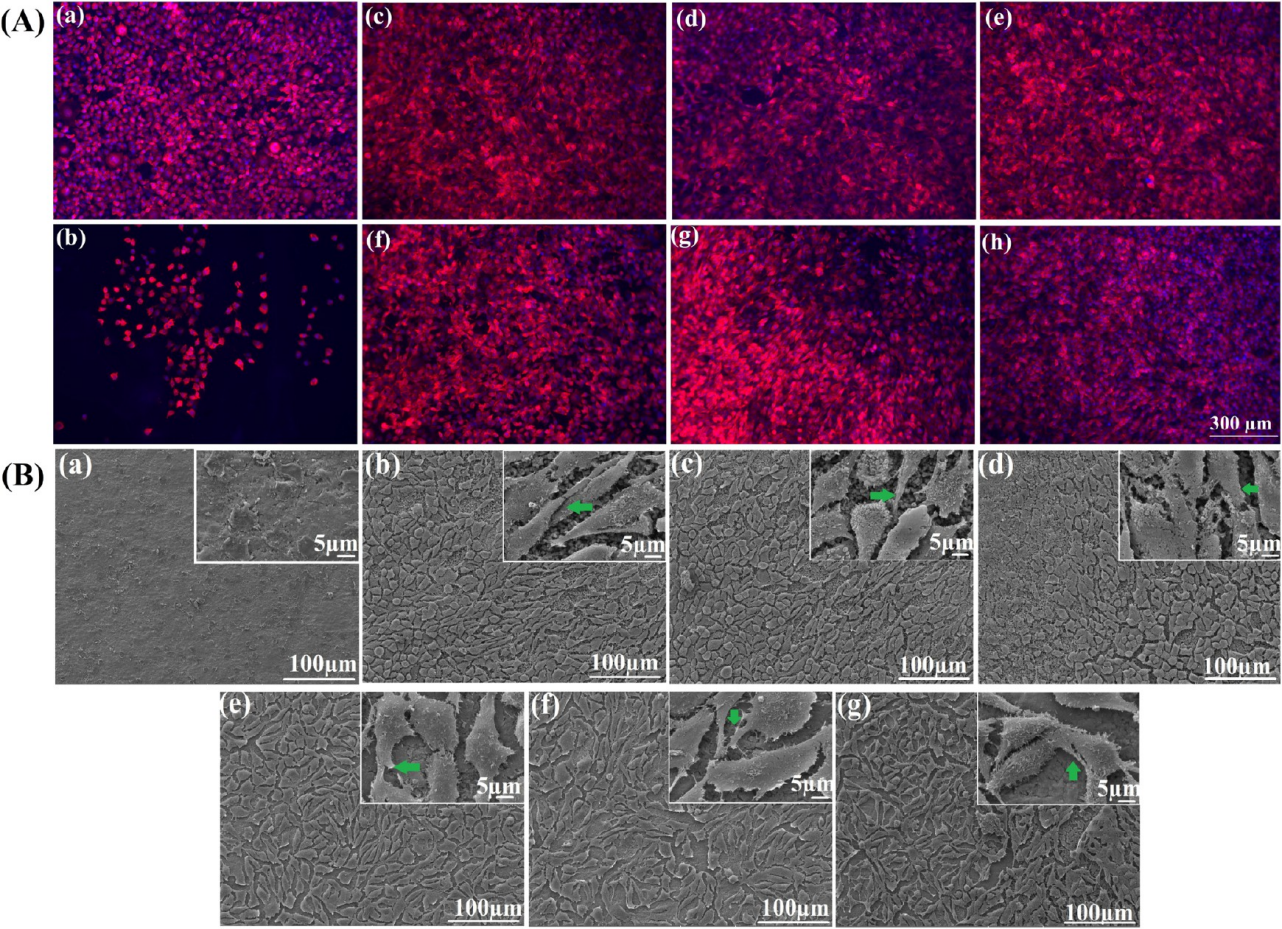
(A) Cytoskeleton imaging of L929 cells treated with (a) blank, (b) APTES, (c–e) control, and (f–h) MGO-incorporated L5, L7, and L9 at 7th day; scale bar: 300 μm; (B) L929 cell adhesion examination by SEM analysis for (a) APTES, (b–d) control, and (e–g) MGO-incorporated L5, L7, and L9 at 7th day, the filopodia of cells was denoted by a green arrow. Scale bar corresponds to 100 μm, and inset images scale bar corresponds to 10 μm.

**Figure 8:**
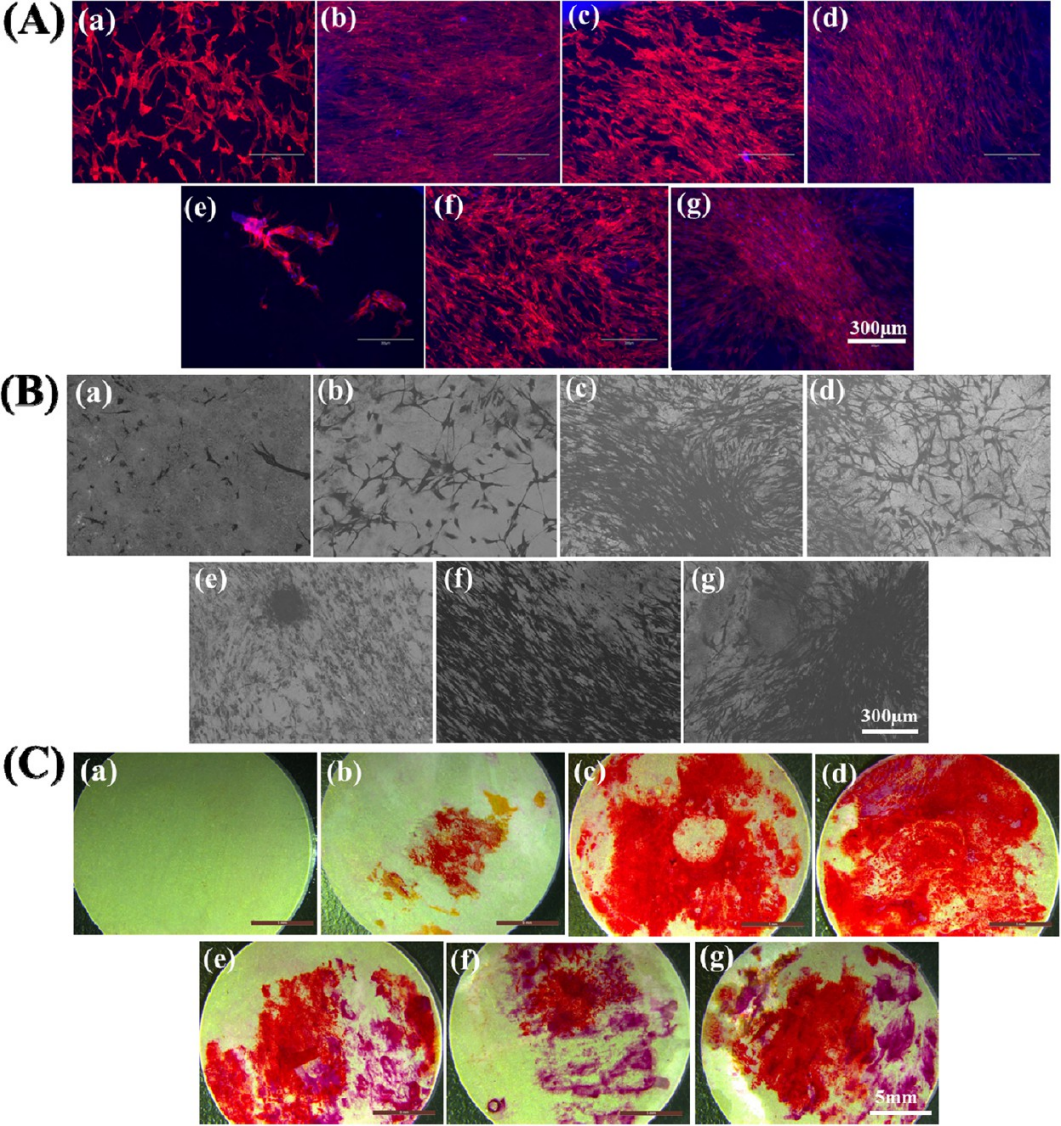
*In vitro* Y201 MSCs cellular interactions. (A) DAPI/phalloidin fluorescence imaging and (B) SEM images. (C) Alizarin Red staining images of (a) APTES-Ti64, (b–d) control. and (e–g) MGO-incorporated L5, L7. and L9 at 7th day.

To examine the cytocompatibility behavior of developed MGO incorporated into L5, L7, and L9 samples, we used two types of cells: L929 fibroblasts and Y201 immortalized human stem cells. Both cells were found to have responsive interactions with our samples, as evidenced by the data in Figures 6–8.

#### 3.9.1 Metabolic Activity of Fibroblast (L929) Cells onto MGO-Loaded Samples

The cellular metabolic activity of L929 cells was assessed by the PrestoBlue assay against the tissue culture plate (TCP), APTES-Ti64, control, and MGO-loaded samples for 1, 3, and 7 days. From the PrestoBlue results (Figure 6A), statistically significant increases in cellular metabolic activity were seen over the period of incubation (from 1 to 7 days) for the different samples. The TCP showed a higher proliferation rate of L929 cells at all time points when compared to the rest of the samples. The cells seeded onto the control samples (without MGO) such as L5, L7, and L9 evidenced a higher metabolic activity (86–89%, *p* < 0.05) when compared to the APTES-Ti64 (41%, *p* < 0.05). MGO-loaded L5, L7, and L9 layers demonstrated suitable metabolic activity of L929 cells (93–96%, *p* < 0.05) at each time point and found to have no statistically significant difference when compared with control samples. However, the control and MGO-loaded samples showed incremental L929 cellular activity with an increase in treating periods from 1 to 7 days (42–96%, *p* < 0.05). From these data, it can be confirmed that the presence of MGO within the HA/J-COLL multilayered coating did not affect the cellular metabolism of L929 fibroblasts, evidencing a suitable cytocompatibility of the multilayers containing MGO.

#### 3.9.2 Viability Assessment via Live/Dead Assay

Qualitative examination of L929 cellular interactions with APTES-Ti64, control, and MGO-loaded samples was evaluated by fluorescence-based live/dead assays at 1 and 7 days (Figure 6B). On day 1, it was noticed that all of the sample surfaces showed minimal covering of cells with good cell viability along with staining of a blue color, which indicates the live cells. On day 7, the control and MGO-loaded layers showed better cell expansion on their surfaces than APTES-Ti64. The control and MGO-loaded sample surfaces were completely covered by L929 cells, and their growth was found to be strong with no qualitative significant difference. The thick cell monolayer was deposited on the surface of samples with an increase in time point from 1 to 7 days. The seeded cells appeared mostly stained in blue color, which indicates a higher number of live cells, and very few/no green stained cells were observed evidencing very few/no dead cells. These results were consistent with metabolic activity results and confirmed MGO-loaded layers supporting suitable and proper cell viability.

#### 3.9.3 Morphological Analysis of Fibroblast (L929) Attachment onto Coated Samples

The actin and nuclei of L929 cells were stained with phalloidin (red) and DAPI (blue), respectively, on the surface of APTES-Ti64, control, and MGO-loaded samples on day 7 (Figure 7A). From the images, it was observed that TCP and APTES-Ti64 presented a round-shaped morphology with minimal expansion. In contrast, cells seeded onto multilayered samples with and without MGO appeared uniformly distributed onto the substrates, displaying elongated polygonal shape morphology and proper cellular spreading onto the substrates. In addition, L929 cells’ cytoskeleton presented an expansion of actin protein filament with points of focal adhesion (stained in red color and nucleus in blue color). These results demonstrate that multilayered samples, with and without MGO, promoted cellular attachment and growth, visually supporting the results obtained by PrestoBlue and live/dead assays.

Moreover, on day 7, we used SEM analysis to examine the attachment of L929 cells on the surface of APTES-Ti64, multilayered samples with and without MGO. From SEM images (Figure 7B), it was observed that APTES-Ti64 revealed less attachment of L929 cells, whereas multilayered samples with and without MGO samples displayed a higher amount of cellular adhesion with homogeneous distribution over their surfaces. The shape of the cells was found to have a spindle-like structure and extended filopodia, fully adhered to the multilayered substrates with and without MGO. SEM analysis evidenced that MGO-incorporated layers supported the L929 cells’ attachment.

#### 3.9.4 Impact of MGO-Loaded HA/J-COLL Coating on Immortalized Stem Cells (TERT-hMSCs, Y201)

The cytoskeletal structure of Y201 hMSCs was examined on day 7 for APTES-Ti64, multilayered (L5, L7, and L9) samples with and without MGO (Figure 8A). The multilayered samples without MGO supported the growth of Y201 with a spindle-shaped morphology, whereas APTES-Ti64 has a slight spread of cells on its surface. The cells seeded onto MGO-loaded sample surfaces displayed elongated filament structures; however, the MGO-loaded L5 layer suggested less growth of cells, and they appeared highly clustered. The immunostaining images confirm that the MGO agent in different layers of HA/J-COLL facilitates the growth of Y201 cells for L7 and L9. SEM images (Figure 8B) demonstrate the attachment of Y201 cells on the surface of all samples on day 7. In contrast, the multilayered samples with and without MGO showed comparable cellular attachment, spreading, and confluence with no significant difference.

The Y201 cells’ differentiation and deposition of calcium minerals onto multilayered samples with and without MGO were studied by the qualitative alizarin red staining (ARS) method. From ARS images (Figure 8C), the multilayered samples with and without MGO showed a red color staining of deposited calcium mineral, whereas APTES-Ti64 did not show any red staining. On day 7, it was noted that MGO-loaded samples displayed less red staining than the control samples due to a lower amount of calcium mineral deposition. The multilayered samples without MGO showed higher calcium deposition due to the presence of HA and J-COLL.

## 4 Discussion

In this study, for the first time, to improve the anti-infection efficacy of HA/J-COLL multilayer coatings, we explored the potential of the Manuka honey-derived MGO antibacterial agent by incorporating it in multilayers of HA/J-COLL coatings developed on Ti64 by our customized hybrid LbL machine. The MGO-loaded HA/J-COLL coatings significantly reduced *E. coli* and *S. epidermidis* bacterial growth and colonization on their surface. The cytocompatibility tests revealed that the MGO-loaded coatings did not affect cellular metabolism and supported cellular attachment, growth, and differentiation of L929 fibroblasts; also, the MGO-loaded coatings differentiated Y201 cells and secreted a minimal amount of calcium minerals on their surface.

Our team created a hybrid layer-by-layer automated machine (US Patent: US20220388024A1) for thin-film fabrication and layer-by-layer deposition on metal and polymeric substrates. To deposit a layer on a substrate, this instrument includes the processes of (i) partially submerging the substrate in a reservoir of a charged solution and (ii) spraying the substrate with an atomized charged solution. When compared with conventional techniques, our LbL equipment shortens the overall duration of coating growth. In one instance, 20 nanolayers can be produced in 15–0 min rather than in 350–400 min using the traditional immersive LbL method.^37^ The surface of Ti64 substrates was positively activated by ammonium ions (NH_4_^+^) to favor the electrostatic deposition of oppositely charged polyelectrolytes. The ζ-potential results confirmed the surface charges of J-COLL (2 mg/mL), HA (1 mg/mL), and MGO (0.625 mg/mL) as +3.11, −29.36, and −3.08 mV, respectively. From the literature, it was found that the surface charge on J-COLL was not yet reported, but our study strived to confirm the positive charge on J-COLL, which is analogous to the ζ-potential value of 2.5 mV of mammalian-derived COLL (rat tail tendon)reported by Niu et al.^38^ The self-assembling interactions between HA and J-COLL were confirmed by the typical trend of zigzag contact angle values, and the electrostatic interactions were strong up to the ninth layer deposition of HA. Our reported contact angle values of J-COLL and HA were substantiated by Zhao et al.’s^30^ report on hydrophilic (30°) and hydrophobic (110°) values of HA and COLL, respectively. In optimized self-assembly multilayers, we have selected L5, L7, and L9 to include the MGO compound. However, the control and MGO-included samples have good hydrophilicity because the main composition of the terminal layer is the HA material. The surface roughness of HA/J-COLL multilayer coatings with and without MGO showed statistically no significance of *R*_a_ values in the range of *R*_a_ = 1.1032–1.3466 μm, whereas the surface morphology results demonstrated dense coatings with the accumulation of HA/J-COLL multilayers. The incorporation of the MGO compound into multilayers did not influence the surface roughness and morphology due to well-ordered integration in multilayers. ATR-FTIR spectra confirmed the inclusion of the MGO compound into multilayers via a minor lower shift of the vibrational band at 1644 cm^–1^, which indicates the presence of the keto group (C=O) in the MGO and matches with the literature.^35^ XPS analysis confirmed deposition of HA and J-COLL molecules and that the atomic concentrations of N 1s and C 1s were higher than APTES-Ti64. The N 1s and C 1s are major elements of HA and J-COLL, which provide strong evidence of multilayer deposition on Ti64 and are substantiated by previous reports.^8^ The MGO was quantified at 6.60–7.82 mM, which matched the actual included MGO (9 mM) in multilayers, and the releasing profile was found to be controlled for up to 21 days.

The MIC and MBC values of MGO against *E. coli* and *S. epidermidis* were similar to an existing report^36^ and the minimum concentration of MGO required to kill both bacteria was estimated as 9 mM. It is well-known from the literature that when implantation occurs in the body, early-stage infections can occur, and treating them with antibiotics is difficult if they persist in forming biofilms.^39,40^ In our work, we explored two strategies: (i) the release of MGO for the prevention of bacteria and (ii) the contact-killing of bacteria. Therefore, we have included MGO in the top layers (L5―1×, L7―2×, and L9―3×) to enhance the bacterial killing efficacy of the HA/J-Coll LbL coating through the release of MGO and contact-killing. The planktonic and biofilm bacterial quantification by viable counts confirmed that MGO-loaded layers 7 and 9 have a favorable effect on preventing *E. coli* and *S. epidermidis* bacterial growth and inhibit their colonization on the surfaces. It is important to note that these assays were performed in rich growth media, where bacterial growth is likely to outpace the accumulation of MGO in the medium. Therefore, we would not expect large reductions, particularly in planktonic cell growth. Nevertheless, significant reductions in the growth of bacteria were observed and were supported by qualitative assessments by microscopy (SEM and CLSM). In the human body, bacterial growth will be far slower, providing additional time for MGO to accumulate and aid the immune system in clearing bacterial cells. Animal models will be needed to determine the impact of these membranes on microbial growth under more realistic conditions.

These antibacterial studies showed that MGO was released from the multilayers in a controlled manner to kill both planktonic bacteria and support the contact-killing behavior to prevent the adhesion and colonization of biofilm bacteria. Our reported antibacterial results of MGO-loaded HA/J-COLL coatings agreed with the report by Haiyong et al. on the contact and release killing of pathogens.^41^ The Gram-positive bacteria (*S. epidermidis*) possess a cell membrane and a thick layer of peptidoglycan, while Gram-negative bacteria (*E. coli*) have an inner cell membrane, peptidoglycan layer, and outer membrane. Bacteria are shielded by these layers from the hostile extracellular environment. The proteins of the flagella and fimbriae, on the other hand, come into contact with the environment. MGO can bind directly to fimbriae and flagellar proteins including FimA/PapA and flagellin, causing loss of structural integrity and subsequently function in addition to altering gene expression and flagellum motility.^42,43^ Also, due to its electrophilic nature, MGO can bind to DNA and protein chemical moieties such as guanine, arginine, lysin, etc. than alter their synthetic derivatives, structures, and functions.^44,45^ Roberts et al. demonstrated that manuka honey prevented the growth of bacteria by constraining flagella-allied genes.^46^ Rabie et al. observed that MGO at a concentration of 2 mM causes the loss of *E. coli*, *Pseudomonas aeruginosa*, *Staphylococcus aureus*, *Bacillus subtilis* bacterial fimbriae and flagella in addition to damaging cell membranes, shrinkage, and rounding of bacterial cells.^47^ Few studies demonstrated the effectiveness of MGO toward methicillin- and oxacillin-resistant *S. aureus*.^48,49^ According to Kilty et al., MGO was efficient against methicillin-resistant *S. aureus* (MRSA) biofilms *P. aeruginosa* and *S. aureus*. The effective concentrations of MGO for planktonic MRSA and *P. aeruginosa* were found to be 1.1–4.16 and 2.08–16.65 mM, respectively, whereas for biofilms, the MRSA was significantly greater than for planktonic bacteria (6.94–50.0 and 24.98–101.30 mM).^50^ However, *in vivo* tests, such as percutaneous and subcutaneous infection studies, are highly needed to understand the impact of MGO on bacterial elimination by either protein or gene damage.

*In vitro* cytocompatibility studies of multilayers with and without MGO demonstrated no toxicity toward L929 fibroblast cells; however, the cellular metabolic activity was found to increase for control and MGO-loaded multilayers than APTES-Ti64, and the activity was gradually increased with an increase in time points from 1 to 7 days. The live/dead images of control and MGO-loaded multilayers displayed the thick monolayer of cellular growth on their surfaces with active cells from days 1 to 7. The cytoskeleton staining and SEM images of control and MGO-loaded multilayers on day 7 showed that L929 cells had an elongated polygonal shape morphology with the expansion of actin protein filament and focal adhesion. The reason behind the improvement of L929 cellular functions is due to the physicochemical properties of multilayer coatings including surface roughness, morphology, wettability, and chemical composition. The surface roughness and morphology were found to be similar for control and MGO-loaded multilayer coatings in terms of the roughness nature, which are key factors due to deposition of oppositely charged HA and J-COLL, which deliver the appropriate location to adhere the cells, infiltration, and growth. The surface wettabilities of control and MGO-loaded multilayers were found to be in the range of 51.17–66.73°, which is aligned with an earlier report on that the contact angle value of about 60° is optimal wettability for supporting the cellular interactions.^8^

Usually, there are three stages in cellular communications with the surfaces: (i) the attachment of cell numbers increasing with time, (ii) the flattening of cells, and (iii) the elongation of cell structures.^51^ The chemical composition of multilayers was J-COLL and HA, which positively affect the cellular behavior of L929 in terms of adhesion, growth, proliferation, and hMSCs (Y201) differentiation. So far, few reports exist on the usage of marine-derived COLL for biomedical applications, including in bone, cartilage, and industries. Ranny et al. fabricated jellyfish (*Aurelia aurita*)-extracted collagen scaffolds for the regeneration of the alveolar bone. They controlled the degradation of the scaffold by chemical cross-linking and proved their nontoxic nature against hMSCs.^52^ Andrew et al. proved that J-COLL (*Rhizostoma pulmo: R. pulmo*) is a promising next generation of biomaterials and regenerative medicine. They studied the cell adhesion, cell viability, and immunocytochemistry assays of J-COLL against induced pluripotent stem cells (iPSC)-derived microglial-like cells (MGL) and confirmed their superior biofunctions over mammalian-derived collagen (rat tail COLL).^53^ Ahmed et al. found that J-COLL (*R. pulmo*) exhibits promise as a unique, structurally compatible, cleaner, next-generation collagen source that is more reliable for tissue engineering in osteoarthritis healing. J-COLL has structural and biodegradable material qualities, as well as the biocompatibility and immunogenic profiles, necessary for therapeutic usage in OA patients’ cartilage healing.^54^ HA is the outermost component of multilayer coatings, which have strong intrinsic biological functions such as bone-promoting osteogenic, anti-inflammatory, and antibacterial properties. Through controlling Wnt/-Catenin signaling, HA significantly promotes the proliferation and differentiation of stem cells in a dose-dependent manner without affecting the cytophenotype.^55^ Limited research has been conducted on MGO to reveal its antibacterial and anticancer functions,^56,57^ yet a few contradictory reports exist on the bone cells’ interactions with MGO.^58−60^ The role of the MGO in the differentiation of human bone marrow-derived stromal cells (hBMSCs) into osteoblasts is still not clear. However, in our study, the MGO-loaded multilayers did not show potential toxicity toward L929 and Y201 cells due to the lower concentration (9 mM), controlled release behavior, and synergistic effect of their combination with HA and J-COLL biomolecules.

On the other hand, we have evaluated the osteogenic ability of control and MGO-loaded multilayer coatings with Y201 hMSCs at 7 days by qualitative in vitro assays such as cytoskeleton staining, SEM, and ARS staining tests. The immunostaining images displayed the growth of Y201 cells with spindle shape morphology on control and MGO-loaded multilayers, whereas SEM micrographs showed comparable cellular attachment, spreading, and confluence. The ARS staining confirms the calcium mineralization competence of the control and MGO-loaded multilayers and their capability to differentiate Y201 hMSCs into osteoblast cells. Our study is the first report of the influence of J-COLL on the differentiation of Y201 cells and calcium secretion ability. Therefore, these primary results confirm that J-COLL in the coating supported the calcium deposition from the mineralization phase, indicating better differentiation than APTES-Ti64 (uncoated model). In the MGO-loaded samples, there was less presence of calcium compared to the multilayered samples without MGO. There was also evidence that the increased MGO attendance possibly led to a slower increase in the Ca^2+^.^61^ There is still limited literature regarding MGO’s role in the calcification of bone cells, yet research has reported that MGO generates mitochondrial dysfunction in liver cells.^62^ It has been reported that certain collagen amino acids, such as glycine, alanine, glutamine, and asparagine, can selectively induce osteogenesis by activating the focal adhesion kinase/JNK signaling cascade and runt-related transcription factors in bone marrow-derived MSCs.^63^ Rena et al. proved that the enhanced rates of fibril production by tilapia-scale COLL may promote early osteogenic induction via upregulating *alp*, *opn*, and *bmp-2.*(64) Elango et al. studied that hydrolyzed Mahi Mahi fish COLL treated with BMSCs showed significantly elevated expression of the osteogenesis markers *alp*, *coll, and ocn*.^65^ Pugliano et al. developed a novel therapeutic implant for cartilage repair by combining J-COLL (*R. pulmo*), transforming growth factor-3 (TGF-3), and human BMSCs. *In vitro* cellular differentiation results showed that the J-COLL-containing implant prompted chondrogenesis in human MSCs.^66^ However, there is no strong evidence of the impact of J-COLL on osteogenic differentiation and its applications in bone regeneration. In this study, we have successfully proved the antibacterial activity and cytocompatibility of MGO-loaded multilayers, but this work has limitations in terms of broad antibacterial spectrum analysis, and osteogenic and angiogenic evaluation of multilayers with and without MGO. In the future work, we will extend our developed MGO-loaded multilayer coatings toward treating *in vivo* infection models and regenerating animal calvarial bone defects.

## 5 Conclusions

For the first time, multilayered coatings based on J-COLL and HA have been manufactured via the layer-by-layer method on Ti64 alloy substrates by incorporating the MGO compound in the outermost layers. A number of HA/J-COLL multilayers were successfully optimized toward building-up stable electrostatic interactions, evidencing the coatings have strong electrostatic interactions up to the ninth layer. Planktonic and biofilm quantification results confirmed that the MGO concentration in layers 7 and 9 has a favorable effect on preventing bacterial growth and colonization on their surfaces. Moreover, this work has demonstrated that the incorporation of MGO into the multilayered coatings prevented the growth of *E. coli* and *S. epidermidis*, being favorable for contact-killing of both bacterial strains. An *in vitro* cytocompatibility study confirmed that MGO agents including HA/Coll layers did not affect or reduce the cellular metabolism of L929 fibroblasts, demonstrating the suitable cytocompatibility of MGO-incorporated L5, L7, and L9 samples. Indeed, the cytocompatibility tests evidenced that MGO-loaded HA/J-COLL layers sustained cellular attachment and growth of L929 and Y201 cells. Moreover, the MGO-loaded sample supported TERT-hMSCs (Y201) differentiation and mineral deposition after 7 days of incubation as detected by ARS. Therefore, the outcomes from this work show how the use of a MGO natural antibacterial agent incorporated within HA/J-COLL coatings on Ti64 substrates can act as an ideal antimicrobial coating and potential alternative to prevent antimicrobial resistance while favoring bone-inducing functions suitable for metallic orthopedic implants and bone tissue engineering applications.

## Supporting Information Available

Acridine Orange quantification of amine groups and staining images of untreated-Ti64, alkali-Ti64, and silanized-Ti64 sample surfaces (Figure S1A,B); profilometry 2D and 3D images of APTES-Ti64, control, and MGO-incorporated L5, L7, and L9 (Figure S2A,B); antibacterial activity of MGO component and determination of its MIC and MBC using *E. coli* and *S. epidermidis* bacteria (Figure S3A–C); spread plate method used to determine *E. coli* and *S. epidermidis* planktonic viable bacteria on the surfaces of samples for 24 h (Figure S4); spread plate method used to determine *E. coli* and *S. epidermidis* biofilm viable bacteria on the surface of samples for 24 h (Figure S5); and quantification of mean fluorescence intensities of *E. coli* and *S. epidermidis* from CLSM images by ImageJ software (Figure S6) (PDF)

## References

[ref1] Garg D.; Matai I.; Sachdev A. Toward designing of anti-infective hydrogels for orthopedic implants: from lab to clinic. ACS Biomater. Sci. Eng. 2021, 7, 1933–1961. 10.1021/acsbiomaterials.0c01408. 33826312

[ref2] Wu Z.; Chan B.; Low J.; Chu J. J. H.; Hey H. W. D.; Tay A. Microbial resistance to nanotechnologies: An important but understudied consideration using antimicrobial nanotechnologies in orthopaedic implants. Bioact. Mater. 2022, 16, 249–270. 10.1016/j.bioactmat.2022.02.014. 35415290 PMC8965851

[ref3] Sui J.; Liu S.; Chen M.; Zhang H. Surface Bio-Functionalization of Anti-Bacterial Titanium Implants: A Review. Coatings 2022, 12, 1125 10.3390/coatings12081125.

[ref4] Nouri A.; Shirvan A. R.; Li Y.; Wen C. Surface modification of additively manufactured metallic biomaterials with active antipathogenic properties. Smart Mater. Manuf. 2023, 1, 100001 10.1016/j.smmf.2022.100001.

[ref5] Aggarwal D.; Kumar V.; Sharma S. Drug-loaded biomaterials for orthopedic applications: A review. J. Controlled Release 2022, 344, 113–133. 10.1016/j.jconrel.2022.02.029. 35240227

[ref6] Spirescu V. A.; Chircov C.; Grumezescu A. M.; Vasile B. Ş.; Andronescu E. Inorganic nanoparticles and composite films for antimicrobial therapies. Int. J. Mol. Sci 2021, 22, 4595 10.3390/ijms22094595. 33925617 PMC8123905

[ref7] Drexelius M. G.; Neundorf I. Application of antimicrobial peptides on biomedical implants: Three ways to pursue peptide coatings. Int. J. Mol. Sci 2021, 22, 13212 10.3390/ijms222413212. 34948009 PMC8703712

[ref8] Ferreira A. M.; Gentile P.; Toumpaniari S.; Ciardelli G.; Birch M. A. Impact of collagen/heparin multilayers for regulating bone cellular functions. ACS Appl. Mater. Interfaces 2016, 8, 29923–29932. 10.1021/acsami.6b09241. 27762547

[ref9] Gentile P.; Ferreira A. M.; Callaghan J. T.; Miller C. A.; Atkinson J.; Freeman C.; Hatton P. V. Multilayer nanoscale encapsulation of biofunctional peptides to enhance bone tissue regeneration *in vivo*. Adv. Healthcare Mater. 2017, 6, 1601182 10.1002/adhm.201601182. 28169513

[ref10] Udduttula A.; Li J.; Ma Z.; Teng B.; Zhang J. V.; Ferreira A. M.; Ren P. G.; et al. A novel apatite-inspired Sr5 (PO4) 2SiO4 plasma-sprayed coating on Ti alloy promoting biomineralization, osteogenesis and angiogenesis. Ceram. Int. 2022, 48, 10979–10989. 10.1016/j.ceramint.2021.12.317.

[ref11] Gentile P.; Ghione C.; Ferreira A. M.; Crawford A.; Hatton P. V. Alginate-based hydrogels functionalised at the nanoscale using layer-by-layer assembly for potential cartilage repair. Biomater. Sci. 2017, 5, 1922–1931. 10.1039/C7BM00525C. 28752866

[ref12] Cen L.; Liu W. E. I.; Cui L. E. I.; Zhang W.; Cao Y. Collagen tissue engineering: development of novel biomaterials and applications. Pediatr. Res. 2008, 63 (5), 492–496. 10.1203/PDR.0b013e31816c5bc3. 18427293

[ref13] Kosiński J.; Jarecki J.; Przepiórka-Kosińska J.; Ratajczak M. Hyaluronic acid in orthopedics. Wiad Lek 2020, 73, 1878–1881. 10.36740/WLek202009114. 33099534

[ref14] Kim J. E.; Sykes J. M. Hyaluronic acid fillers: history and overview. Facial plast. surg. 2011, 27, 523–528. 10.1055/s-0031-1298785. 22205525

[ref15] Ao H.; Zong J.; Nie Y.; Wan Y.; Zheng X. An *in vivo* study on the effect of coating stability on osteointegration performance of collagen/hyaluronic acid multilayer modified titanium implants. Bioact. Mater. 2018, 3, 97–101. 10.1016/j.bioactmat.2017.07.004. 29744446 PMC5935658

[ref16] Luo Q.; Huang Y.; Deng X.; Zhang J.; Li X.; Zhao S.; Li X. Polyelectrolyte multilayer coating with two regulatory molecules on titanium: construction and its biological effects. Nanomedicine 2013, 8, 739–755. 10.2217/nnm.12.151. 23384699

[ref17] Wu C.; Shao X.; Lin X.; Gao W.; Fang Y.; Wang J. Surface modification of titanium with collagen/hyaluronic acid and bone morphogenetic protein 2/7 heterodimer promotes osteoblastic differentiation. Dent. Mater. J. 2020, 39, 1072–1079. 10.4012/dmj.2019-249. 33028783

[ref18] Huang Y.; Luo Q.; Li X.; Zhang F.; Zhao S. Fabrication and in vitro evaluation of the collagen/hyaluronic acid PEM coating crosslinked with functionalized RGD peptide on titanium. Acta Biomater. 2012, 8, 866–877. 10.1016/j.actbio.2011.10.020. 22040683

[ref19] Li X.; Luo Q.; Huang Y.; Li X.; Zhang F.; Zhao S. The responses of preosteoblasts to collagen/hyaluronic acid polyelectrolyte multilayer coating on titanium. Polym. Adv. Technol. 2012, 23, 756–764. 10.1002/pat.1953.

[ref20] Senadheera T. R.; Dave D.; Shahidi F. Sea cucumber derived type I collagen: A comprehensive review. Mar. Drugs. 2020, 18, 471 10.3390/md18090471. 32961970 PMC7551324

[ref21] Alvarez-Suarez J. M.; Gasparrini M.; Forbes-Hernández T. Y.; Mazzoni L.; Giampieri F. The composition and biological activity of honey: a focus on Manuka honey. Foods 2014, 3, 420–432. 10.3390/foods3030420. 28234328 PMC5302252

[ref22] Talukdar D.; Ray S.; Ray M.; Das S. A brief critical overview of the biological effects of methylglyoxal and further evaluation of a methylglyoxal-based anticancer formulation in treating cancer patients. Drug Metab. Drug Interact. 2008, 23, 175–210. 10.1515/DMDI.2008.23.1-2.175. 18533369

[ref23] Khan S. H.; Younus H.; Allemailem K. S.; Almatroudi A.; Alrumaihi F.; Alruwetei A. M.; Khan M. A. Potential of methylglyoxal-conjugated chitosan nanoparticles in treatment of fluconazole-resistant *Candida albicans* infection in a murine model. Int. J. Nanomed. 2020, 3681–3693. 10.2147/ijn.s249625. PMC7261666 32547022

[ref24] Chakrabarti A.; Talukdar D.; Pal A.; Ray M. Immunomodulation of macrophages by methylglyoxal conjugated with chitosan nanoparticles against Sarcoma-180 tumor in mice. Cell. Immunol. 2014, 287, 27–35. 10.1016/j.cellimm.2013.11.006. 24368179

[ref25] Yang M.; Ward J.; Choy K. L. Nature-inspired bacterial cellulose/methylglyoxal (BC/MGO) nanocomposite for broad-spectrum antimicrobial wound dressing. Macromol. Biosci. 2020, 20, 2000070 10.1002/mabi.202000070. 32567254

[ref26] Yuen A.; Laschinger C.; Talior I.; Lee W.; Chan M.; Birek J.; McCulloch C. A.; et al. Methylglyoxal-modified collagen promotes myofibroblast differentiation. Matrix Biol. 2010, 29, 537–548. 10.1016/j.matbio.2010.04.004. 20423729

[ref27] Gentile P.; Frongia M. E.; Cardellach M.; Miller C. A.; Stafford G. P.; Leggett G. J.; Hatton P. V. Functionalised nanoscale coatings using layer-by-layer assembly for imparting antibacterial properties to polylactide-co-glycolide surfaces. Acta Biomater. 2015, 21, 35–43. 10.1016/j.actbio.2015.04.009. 25871538

[ref28] Sánchez-Bodón J.; Andrade del Olmo J.; Alonso J. M.; Moreno-Benítez I.; Vilas-Vilela J. L.; Pérez-Álvarez L. Bioactive coatings on titanium: a review on hydroxylation, self-assembled monolayers (SAMs) and surface modification strategies. Polymers 2022, 14, 165 10.3390/polym14010165. PMC8747097 35012187

[ref29] Lu K. W.; Lin Y. T.; Wei H. S.; Kuo C. C. Super hydrophilic Modification of Polycarbonate Substrate Surface by Organic Plasma Polymerization Film. Materials 2022, 15, 4411 10.3390/ma15134411. 35806536 PMC9267533

[ref30] Zhao M. Y.; Li L. H.; Li B.; Zhou C. R. LBL coating of type I collagen and hyaluronic acid on aminolyzed PLLA to enhance the cell-material interaction. Express Polym. Lett. 2014, 8, 322–335. 10.3144/expresspolymlett.2014.36.

[ref31] Ghensi P.; Bettio E.; Maniglio D.; Bonomi E.; Piccoli F.; Gross S.; Tessarolo F.; et al. Dental implants with anti-biofilm properties: a pilot study for developing a new sericin-based coating. Materials 2019, 12, 2429 10.3390/ma12152429. 31366076 PMC6695694

[ref32] Ye S. H.; Johnson C. A. Jr; Woolley J. R.; Murata H.; Gamble L. J.; Ishihara K.; Wagner W. R. Simple surface modification of a titanium alloy with silanated zwitterionic phosphorylcholine or sulfobetaine modifiers to reduce thrombogenicity. Colloids Surf. B 2010, 79, 357–364. 10.1016/j.colsurfb.2010.04.018. PMC3178391 20547042

[ref33] Jia W.; Li M.; Kang L.; Gu G.; Guo Z.; Chen Z. Fabrication and comprehensive characterization of biomimetic extracellular matrix electrospun scaffold for vascular tissue engineering applications. J. Mater. Sci. 2019, 54 (15), 10871–10883. 10.1007/s10853-019-03667-6.

[ref34] Ao H.; Xie Y.; Tan H.; Yang S.; Li K.; Wu X.; Zheng X.; Tang T. Fabrication and in vitro evaluation of stable collagen/hyaluronic acid biomimetic multilayer on titanium coatings. J. R. Soc., Interface 2013, 10, 20130070 10.1098/rsif.2013.0070. 23635490 PMC3673146

[ref35] Ellerbrock R. H.; Gerke H. H. FTIR spectral band shifts explained by OM–cation interactions. J. Plant Nutr. Soil Sci. 2021, 184, 388–397. 10.1002/jpln.202100056.

[ref36] Juliano C.; Magrini G. A. Methylglyoxal, the major antibacterial factor in manuka honey: an alternative to preserve natural cosmetics?. Cosmetics 2019, 6, 1 10.3390/cosmetics6010001.

[ref37] Ferreira A. M.; Gentile P. Thin film fabrication method and apparatus US PatentUS2022/0388024A1, 2022.

[ref38] Niu L. N.; Jee S. E.; Jiao K.; Tonggu L.; Li M.; Wang L.; Tay F. R.; et al. Collagen intrafibrillar mineralization as a result of the balance between osmotic equilibrium and electroneutrality. Nat. Mater. 2017, 16, 370–378. 10.1038/nmat4789. 27820813 PMC5321866

[ref39] Khatoon Z.; McTiernan C. D.; Suuronen E. J.; Mah T. F.; Alarcon E. I. Bacterial biofilm formation on implantable devices and approaches to its treatment and prevention. Heliyon 2018, 4 (12), e01067 10.1016/j.heliyon.2018.e01067. 30619958 PMC6312881

[ref40] Li P.; Yin R.; Cheng J.; Lin J. Bacterial Biofilm Formation on Biomaterials and Approaches to Its Treatment and Prevention. Int. J. Mol. Sci. 2023, 24 (14), 11680 10.3390/ijms241411680. 37511440 PMC10380251

[ref41] Ao H.; Yang S.; Fan Q.; Zhang Q.; Zong J.; Guo S.; Tang T.; et al. Improved antibacterial properties of collagen I/hyaluronic acid/quaternized chitosan multilayer modified titanium coatings with both contact-killing and release-killing functions. J. Mater. Chem. B 2019, 7, 1951–1961. 10.1039/C8TB02425A. 32255058

[ref42] Nolan V. C.; Harrison J.; Cox J. A. Dissecting the antimicrobial composition of honey. Antibiotics 2019, 8, 251 10.3390/antibiotics8040251. 31817375 PMC6963415

[ref43] Guttenplan S. B.; Kearns D. B. Regulation of flagellar motility during biofilm formation. FEMS Microbiol. Rev. 2013, 37 (6), 849–871. 10.1111/1574-6976.12018. 23480406 PMC3718880

[ref44] Ferguson G. P.; Tötemeyer S.; MacLean M. J.; Booth I. R. Methylglyoxal production in bacteria: suicide or survival?. Arch. Microbiol. 1998, 170, 209–218. 10.1007/s002030050635. 9732434

[ref45] Yadav S. K.; Singla-Pareek S. L.; Ray M.; Reddy M. K.; Sopory S. K. Methylglyoxal levels in plants under salinity stress are dependent on glyoxalase I and glutathione. Biochem. Biophys. Res. Commun. 2005, 337 (1), 61–67. 10.1016/j.bbrc.2005.08.263. 16176800

[ref46] Roberts A. E. L.; Maddocks S. E.; Cooper R. A. Manuka honey reduces the motility of *Pseudomonas aeruginosa* by suppression of flagella-associated genes. J. Antimicrob. Chemother. 2015, 70 (3), 716–725. 10.1093/jac/dku448. 25404649

[ref47] Rabie E.; Serem J. C.; Oberholzer H. M.; Gaspar A. R. M.; Bester M. J. How methylglyoxal kills bacteria: An ultrastructural study. Ultrastruct. Pathol. 2016, 40, 107–111. 10.3109/01913123.2016.1154914. 26986806

[ref48] Jenkins R. E.; Cooper R. Synergy between oxacillin and manuka honey sensitizes methicillin-resistant *Staphylococcus aureus* to oxacillin. J. Antimicrob. Chemother. 2012, 67, 1405–1407. 10.1093/jac/dks071. 22382468

[ref49] Stewart J. A.; McGrane O. L.; Wedmore I. S. Wound care in the wilderness: is there evidence for honey?. Wilderness Environ. Med. 2014, 25, 103–110. 10.1016/j.wem.2013.08.006. 24393701

[ref50] Kilty S. J.; Duval M.; Chan F. T.; Ferris W.; Slinger R. Methylglyoxal:(active agent of manuka honey) in vitro activity against bacterial biofilms. In. . In International Forum of Allergy & Rhinology; Wiley Subscription Services, Inc., A Wiley Company: Hoboken, 2011; Vol. 1, pp 348–350. 22287464 10.1002/alr.20073

[ref51] Majhy B.; Priyadarshini P.; Sen A. K. Effect of surface energy and roughness on cell adhesion and growth–facile surface modification for enhanced cell culture. RSC Adv. 2021, 11, 15467–15476. 10.1039/D1RA02402G. 35424027 PMC8698786

[ref52] Rachmawati R.; Hidayat M.; Permatasari N.; Widyarti S. Jellyfish (*Aurelia aurita*) collagen scaffolds potential in alveolar bone regeneration. F1000Research 2021, 10, 318 10.12688/f1000research.28402.1).

[ref53] Mearns-Spragg A.; Tilman J.; Tams D.; Barnes A. The biological evaluation of jellyfish collagen as a new research tool for the growth and culture of iPSC derived microglia. Front. Mar. Sci. 2020, 7, 689 10.3389/fmars.2020.00689.

[ref54] Ahmed Z.; Powell L. C.; Matin N.; Mearns-Spragg A.; Thornton C. A.; Khan I. M.; Francis L. W. Jellyfish collagen: A biocompatible collagen source for 3d scaffold fabrication and enhanced chondrogenicity. Mar. Drugs 2021, 19, 405 10.3390/md19080405. 34436244 PMC8400217

[ref55] Agarwal G.; Agiwal S.; Srivastava A. Hyaluronic acid containing scaffolds ameliorate stem cell function for tissue repair and regeneration. Int. J. Biol. Macromol. 2020, 165, 388–401. 10.1016/j.ijbiomac.2020.09.107. 32961192

[ref56] Leone A.; Nigro C.; Nicolò A.; Prevenzano I.; Formisano P.; Beguinot F.; Miele C. The dual-role of methylglyoxal in tumor progression–novel therapeutic approaches. Front. Oncol. 2021, 11, 645686 10.3389/fonc.2021.645686. 33869040 PMC8044862

[ref57] Ghosh S.; Chakraborty P.; Saha P.; Acharya S.; Ray M. Polymer based nanoformulation of methylglyoxal as an antimicrobial agent: efficacy against resistant bacteria. RSC Adv. 2014, 4, 23251–23261. 10.1039/C4RA00075G.

[ref58] Waqas K.; Muller M.; Koedam M.; El Kadi Y.; Zillikens M. C.; Van der Eerden B. C. J. Methylglyoxal–an advanced glycation end products (AGEs) precursor–Inhibits differentiation of human MSC-derived osteoblasts in vitro independently of receptor for AGEs (RAGE). Bone 2022, 164, 116526 10.1016/j.bone.2022.116526. 35995334

[ref59] Takanche J. S.; Kim J. E.; Jang S.; Yi H. K. Insulin growth factor binding protein-3 enhances dental implant osseointegration against methylglyoxal-induced bone deterioration in a rat model. J Periodontal Implant Sci. 2022, 52, 155 10.5051/jpis.2101200060. 35505576 PMC9064780

[ref60] Aikawa T.; Matsubara H.; Ugaji S.; Shirakawa J.; Nagai R.; Munesue S.; Tsuchiya H.; et al. Contribution of methylglyoxal to delayed healing of bone injury in diabetes. Mol. Med. Rep. 2017, 16, 403–409. 10.3892/mmr.2017.6589. 28534951

[ref61] Lai S. W. T.; Lopez Gonzalez E. D. J.; Zoukari T.; Ki P.; Shuck S. C. Methylglyoxal and its adducts: Induction, repair, and association with disease. Chem. Res. Toxicol. 2022, 35, 1720–1746. 10.1021/acs.chemrestox.2c00160. 36197742 PMC9580021

[ref62] Seo K.; Ki S. H.; Shin S. M. Methylglyoxal induces mitochondrial dysfunction and cell death in liver. Toxicol. Res. 2014, 30, 193–198. 10.5487/TR.2014.30.3.193. 25343013 PMC4206746

[ref63] Liu C. Application of marine collagen for stem-cell-based therapy and tissue regeneration (Review). Med. Int. 2021, 1 (3), 6 10.3892/mi.2021.5. PMC9855277 36698868

[ref64] Matsumoto R.; Uemura T.; Xu Z.; Yamaguchi I.; Ikoma T.; Tanaka J. Rapid oriented fibril formation of fish scale collagen facilitates early osteoblastic differentiation of human mesenchymal stem cells. J. Biomed. Mater. Res. A 2015, 103, 2531–2539. 10.1002/jbm.a.35387. 25546439

[ref65] Elango J.; Robinson J.; Zhang J.; Bao B.; Ma N.; De Val J. E. M. S.; Wu W. Collagen peptide upregulates osteoblastogenesis from bone marrow mesenchymal stem cells through MAPK-Runx2. Cells 2019, 8, 446 10.3390/cells8050446. 31083501 PMC6562845

[ref66] Keller L.; Pugliano M. Combined Jellyfish Collagen Type II, Human Stem Cells and Tgf-β3 as a Therapeutic Implant for Cartilage Repair. J. Stem Cell Res. Ther. 2017, 07, 2 10.4172/2157-7633.1000382.

