# Supplementary material for: Layer-by-Layer Coatings of Collagen–Hyaluronic acid Loaded with an Antibacterial Manuka Honey Bioactive Compound to Fight Metallic Implant Infections
Source: ACS Appl Mater Interfaces. 2023 Dec 6;15(50):58119–35. doi: 10.1021/acsami.3c11910 (PMC10739588; doi:10.1021/acsami.3c11910)
Supplement: Supplementary file 1 — am3c11910_si_001.pdf [file am3c11910_si_001.pdf]

Supporting Information

Layer-by-Layer Coatings of Collagen-Hyaluronic acid Loaded with Antibacterial Manuka Honey Bioactive Compound to Fight Metallic Implant Infections

Anjaneyulu Udduttula[1,5], Nicholas Jakubovics[2], Imran Khan[3], Lucia Pontiroli[3], Kenneth S Rankin[4], Piergiorgio Gentile[1*, ‡], and Ana M. Ferreira[1*, ‡]

[1]School of Engineering, Newcastle University, Newcastle Upon Tyne NE1 7RU, United Kingdom

[2]School of Dental Sciences, Faculty of Medical Sciences, Newcastle University, Newcastle Upon Tyne NE1 7RU, United Kingdom

[3] Biomet UK Healthcare Ltd, Stella Building, Windmill Hill Business Park, Swindon, SN5 6NX, United Kingdom

[4]Translational and Clinical Research Institute, Faculty of Medical Sciences, Newcastle University, NE2 4HH, United Kingdom

[5]Centre of Biomaterials, Cellular & Molecular Theranostics (CBCMT), Vellore Institute of Technology (VIT), Vellore, TN, 632014, India

*Corresponding authors, email address: piergiorgio.gentile@newcastle.ac.uk and ana.ferreira-duarte@newcastle.ac.uk

‡These authors contributed equally.

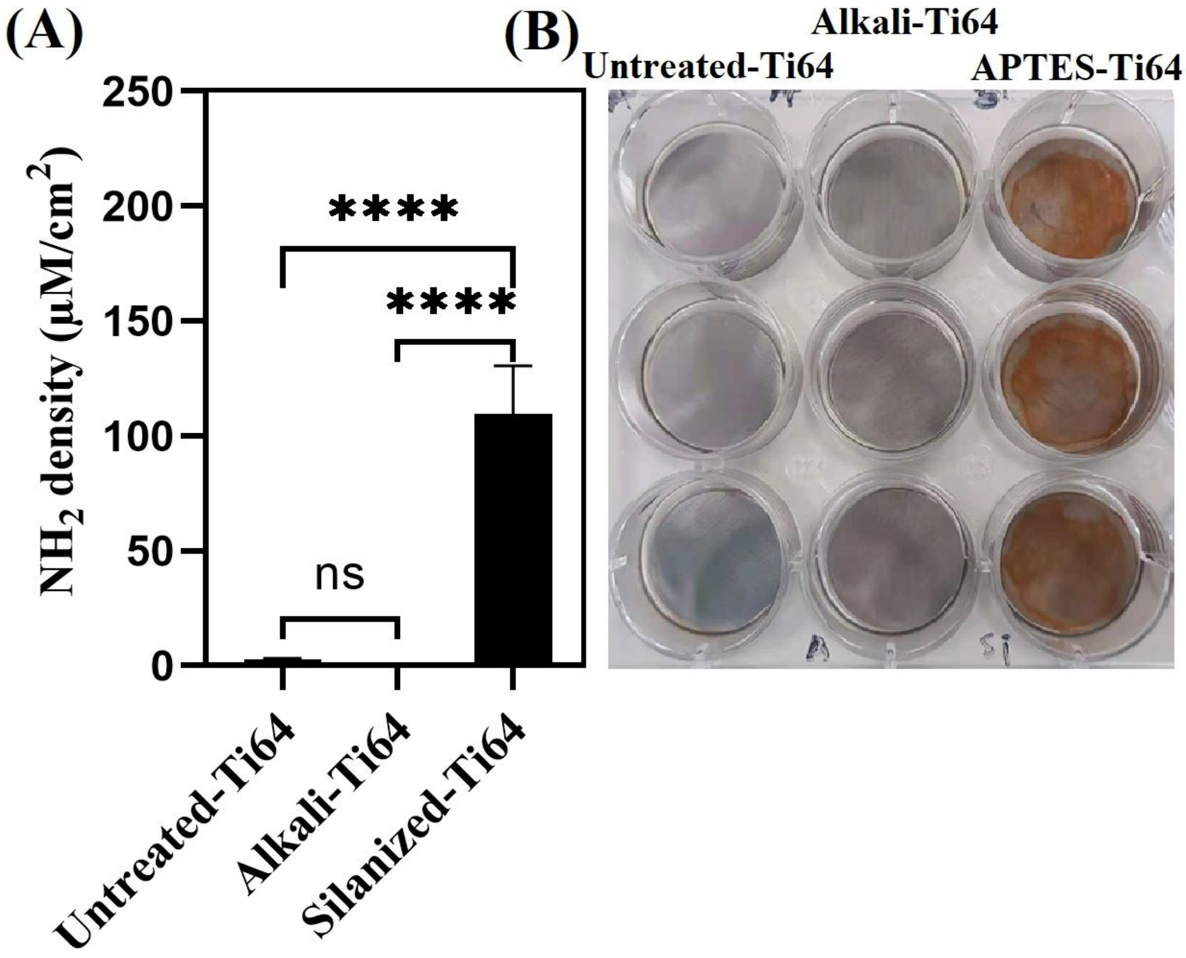

Figure S1. (A) Acridine Orange Quantification of amine groups **(B)** Acridine Orange staining images of untreated-Ti64, alkali-Ti64 and silanized-Ti64 samples surfaces

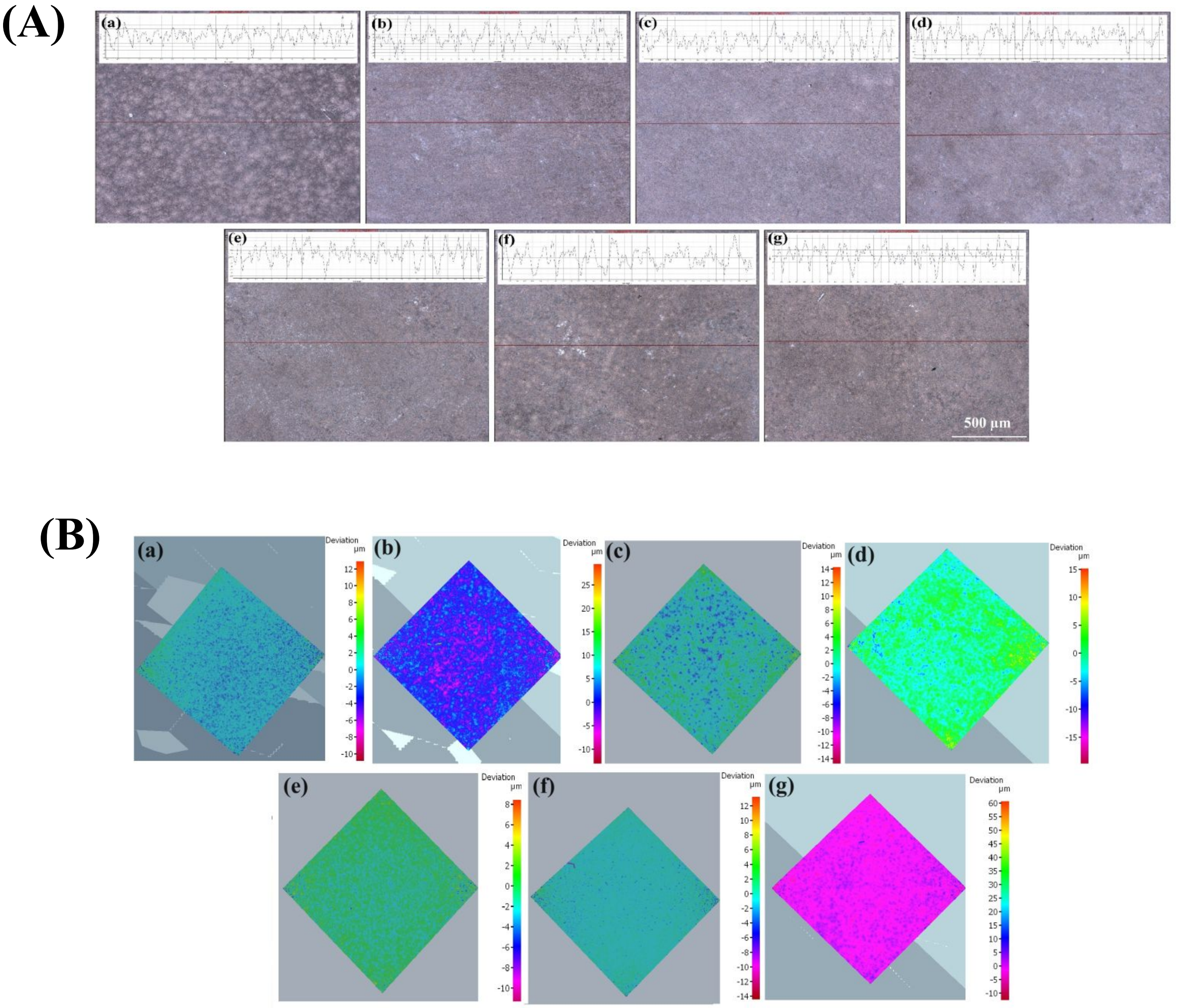

Figure S2. Profilometry (A) 2D and (B) 3D images of (a) APTES-Ti64, (b, c, & d) control and (e, f, &g) MGO incorporated L5, L7 and L9.

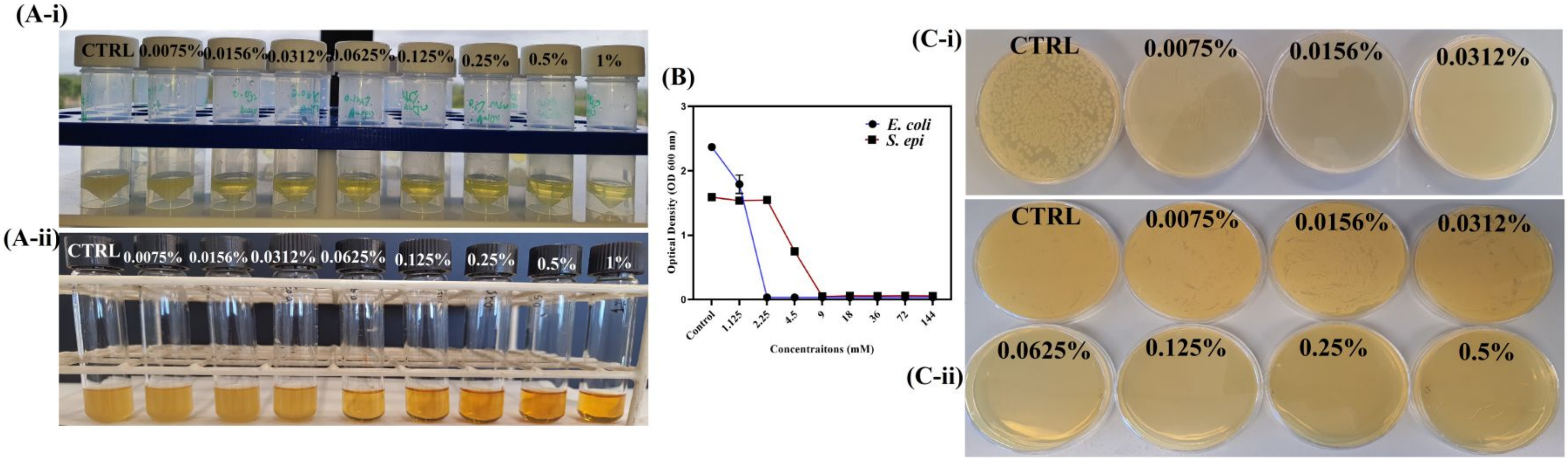

Figure S3. Antibacterial activity of MGO component and determination of its minimum inhibitory concentration (MIC) and minimum bactericidal concentration (MBC) using *E.coli* and *S.epi* bacteria. (A-i) and (A-ii) MIC study of MGO against *E.coli* and *S.epi* respectively, (B) Optical density results of *E.coli* and *S.epi*, (C-i) and (C-ii) MBC study of MGO against *E.coli* and *S.epi* respectively.

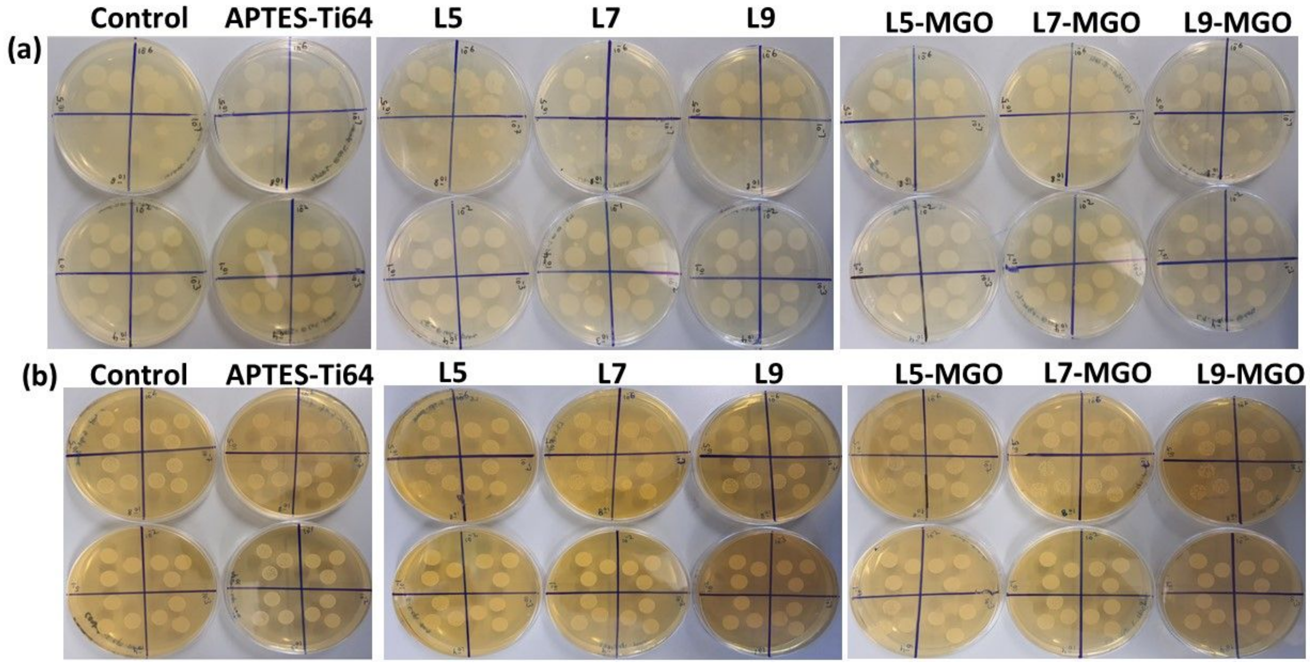

Figure S4. The spread plate method used to determine the *E.coli* and *S.epi* planktonic viable bacteria on the surface of samples for 24 h, (a) *E.coli* bacteria at 10^{-6} (b) *S.epi* bacteria at 10^{-8}

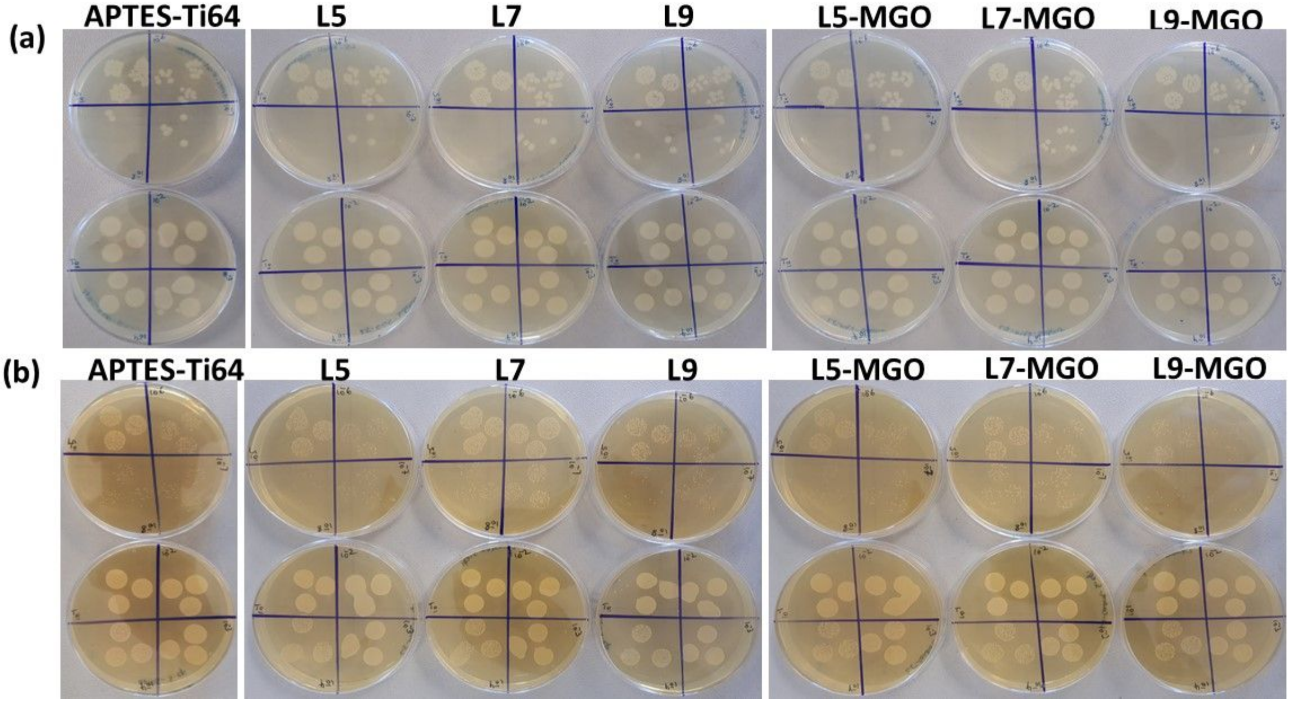

Figure S5. The spread plate method used to determine the *E.coli* and *S.epi* biofilm viable bacteria on the surface of samples for 24 h, (a) *E.coli* bacteria at 10^{-5} (b) *S.epi* bacteria at 10^{-5}

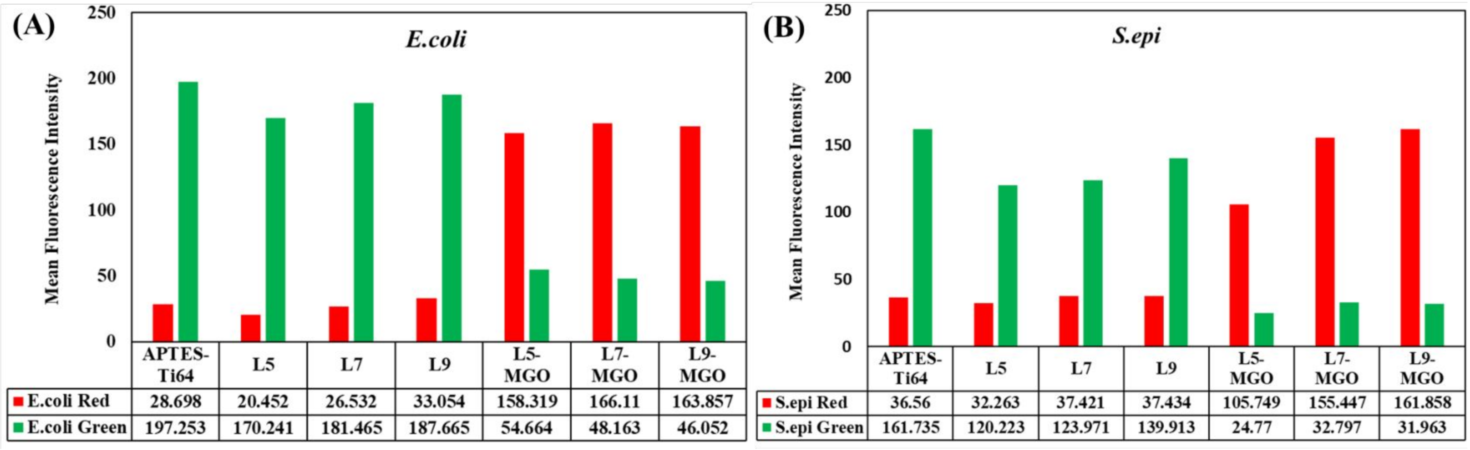

Figure S6. Quantification of mean fluorescence intensities of (A) *E. coli* and (B) *S. epi* from CLSM images by ImageJ software.
